# Desensitization and treatment with APRIL/BLyS blockade in rodent kidney transplant model

**DOI:** 10.1371/journal.pone.0211865

**Published:** 2019-02-08

**Authors:** Natalie M. Bath, Xiang Ding, Nancy A. Wilson, Bret M. Verhoven, Brittney A. Boldt, Adarsh Sukhwal, Shannon R. Reese, Sarah E. Panzer, Arjang Djamali, Robert R. Redfield

**Affiliations:** 1 Department of Surgery, Division of Transplant, University of Wisconsin-Madison, Madison, Wisconsin, United States of America; 2 Department of Medicine, Division of Nephrology, University of Wisconsin-Madison, Madison, Wisconsin, United States of America; Institut Cochin, FRANCE

## Abstract

Alloantibody represents a significant barrier in kidney transplant through the sensitization of patients prior to transplant through antibody mediated rejection (ABMR). APRIL BLyS are critical survival factors for mature B lymphocytes plasma cells, the primary source of alloantibody. We examined the effect of APRIL/BLyS blockade via TACI-Ig (Transmembrane activator calcium modulator cyclophilin lig interactor-Immunoglobulin) in a preclinical rodent model as treatment for both desensitization ABMR. Lewis rats were sensitized with Brown Norway (BN) blood for 21 days. Following sensitization, animals were then sacrificed or romized into kidney transplant **(G4, sensitized transplant control)**; desensitization with TACI-Ig followed by kidney transplant **(G5, sensitized + pre-transplant TACI-Ig)**; kidney transplant with post-transplant TACI-Ig for 21 days **(G6, sensitized + post-transplant TACI-Ig)**; desensitization with TACI-Ig followed by kidney transplant post-transplant TACI-Ig for 21 days **(G7, sensitized + pre- post-transplant TACI-Ig)**. Animals were sacrificed on day 21 post-transplant tissues were analyzed using flow cytometry, IHC, ELISPOT, RT-PCR. Sensitized animals treated with APRIL/BLyS blockade demonstrated a significant decrease in marginal zone non-switched B lymphocyte populations (p<0.01). Antibody secreting cells were also significantly reduced in the sensitized APRIL/BLyS blockade treated group. Post-transplant APRIL/BLyS blockade treated animals were found to have significantly less C4d deposition less ABMR as defined by Banff classification when compared to groups receiving APRIL/BLyS blockade before transplant or both before after transplant (p<0.0001). The finding of worse ABMR in groups receiving APRIL/BLyS blockade before both before after transplant may indicate that B lymphocyte depletion in this setting also resulted in regulatory lymphocyte depletion resulting in a worse rejection. Data presented here demonstrates that the targeting of APRIL BLyS can significantly deplete mature B lymphocytes, antibody secreting cells, effectively decrease ABMR when given post-transplant in a sensitized animal model.

## Introduction

Despite the fact that current one-year kidney allograft survival remains above 90%, little improvement has been made in long-term graft survival.[1] A significant barrier to improving long-term survival in kidney transplant is the lack of effective methods to treat antibody mediated rejection (ABMR) through targeting alloantibody. Alloantibody poses a threat to kidney transplant through two ways: (1) sensitization prior to transplant (2) ABMR. Sensitization occurs through blood transfusions, pregnancy, or prior transplants ultimately results in longer wait-times, increased death on the wait-list, inferior graft outcomes.[2–4] ABMR occurs as a result of preformed alloantibody against the graft or through the development of de novo donor specific antibody (dnDSA).[5–7] Although a multitude of pharmacologic therapies exist to target B lymphocytes at various stages of development, current therapies have failed to effectively treat acute chronic ABMR, which has resulted in a stagnate 10 year graft survival around 50% for patients receiving deceased donor kidney transplants.[1] A long-term solution to ABMR will likely need to focus on multiple targets, which may be achieved through the targeting of APRIL BLyS.

APRIL (a proliferation-inducing lig) BLyS (B lymphocyte stimulator) are members of the tumor necrosis factor (TNF) lig family act as critical survival factors for mature B lymphocytes plasma cells, which are terminally differentiated B lymphocytes. APRIL binds to receptors BCMA (B cell maturation antigen) TACI (Transmembrane activator calcium modulator cyclophilin lig interactor) plays a critical role in plasma cell survival immunoglobulin class switching.[8] BLyS, also known as BAFF (B cell activation factor from the TNF family), also binds to TACI in addition to BAFF-R (BAFF receptor) weakly to BCMA.[9] BLyS provides signals to B lymphocytes for ongoing maturation, proliferation, survival.[10, 11] APRIL BLyS can be targeted through the use of TACI-Ig. TACI-Ig is a recombinant fusion protein that binds neutralizes APRIL BLyS thereby preventing them from binding to their respective receptors.[12]

Here we will explore the efficacy of APRIL/BLyS blockade via TACI-Ig in a sensitized rodent kidney transplant model.

## Materials methods

### Animals

**Fig 1:**
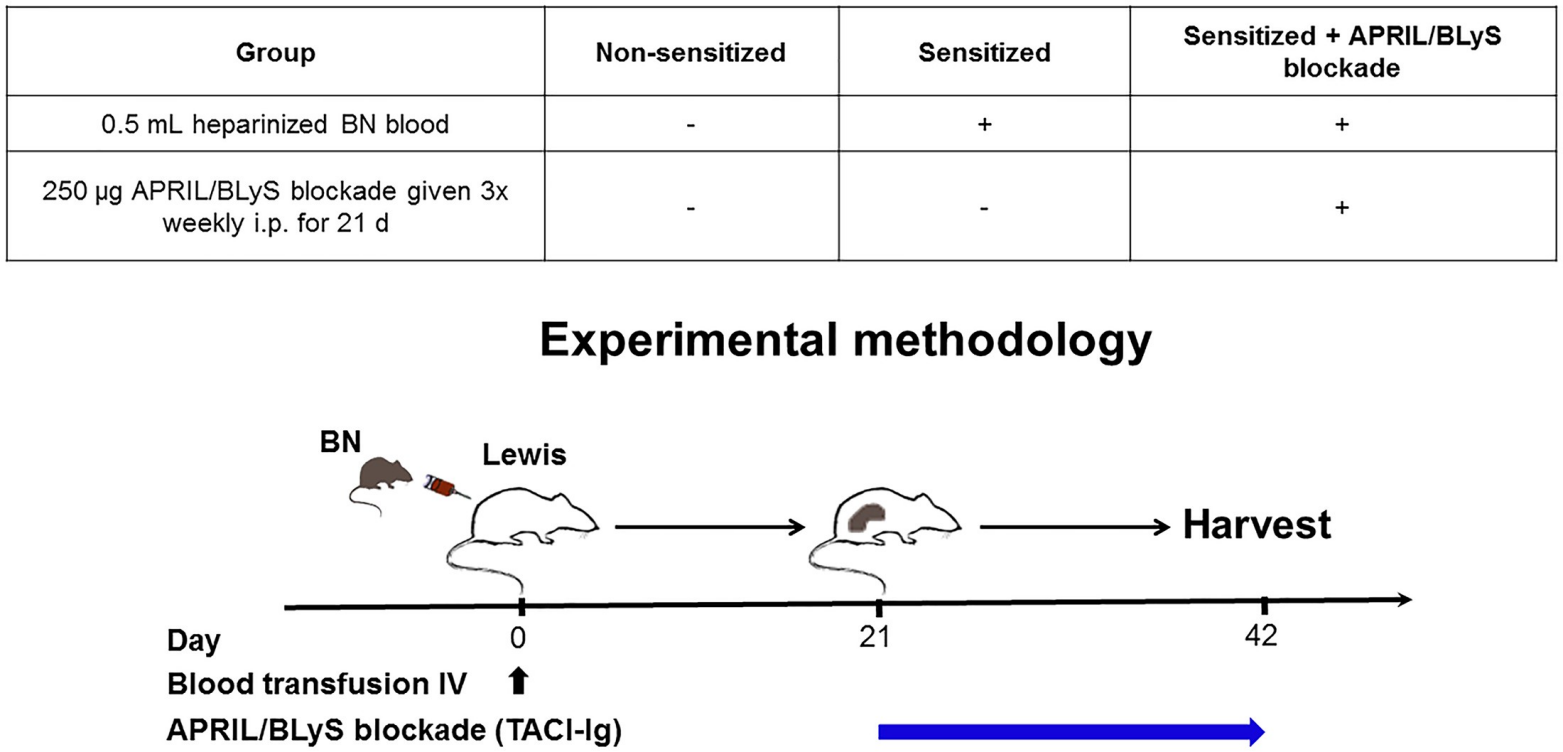
Experimental methodology. Lewis rats were romized into three groups. The non-sensitized group consisted of untreated Lewis rats that were age matched to the other groups (G1, control; N = 4). All other groups were given 0.5mL heparinized blood intravenously from Brown Norway rats. Twenty-one days after sensitization, rats were either left untreated (G2, sensitized group; N = 5) or were given 250 μg APRIL/BLyS blockade (G3, TACI-Ig; N = 6) 3 times weekly for 21 days.

Adult (average 10 weeks) male Lewis (Envigo) adult (average 10 weeks) male Brown Norway (BN) (Envigo) were housed in the University of Wisconsin Laboratory Animal Facility at WIMR. All procedures were performed in accordance with the Animal Care Use Policies at the University of Wisconsin. Animal health including animal deaths, room temperature, 12-hour light/dark cycles, cage cleaning among other sanitation duties were performed daily by WIMR housing staff. Food water were available ad libitum. This research was prospectively approved by School of Medicine Public Health Institutional Animal Care Use Committee at the University of Wisconsin (M005204). Animals that underwent transplantation were monitored daily post-transplant. Animal health was evaluated by activity level, weight gain or loss, hunched posture, other signs of distress. Animals who did not undergo transplant were evaluated using the same criteria three times weekly when injections were administered. For animals undergoing transplant, saline was given IP as needed for dehydration. Lewis rats were sensitized with 0.5 mL heparinized BN blood (complete major histocompatibility complex (MHC) mismatch) given intravenously. Twenty-one days after sensitization animals were then either sacrificed or received TACI-Ig (APRIL/BLyS blockade). APRIL/BLyS blockade was given three times weekly for three weeks after which animals were sacrificed (Fig 1). Animals were anesthetized with isoflurane during surgery or injections sacrificed via cardiac puncture. Buprenorphine (1mg/kg SQ) was used administered post-transplant every 72 hours for the first week following surgery.

APRIL/BLyS blockade was subsequently incorporated into a rodent kidney transplant model. All rodents in this arm of the study were sensitized for 21 days as described above. All rats received cyclosporine (CSA) at the time of transplant in order to minimize cellular rejection until sacrifice. Following sensitization, Lewis rats underwent kidney transplantation from a donor BN bilateral nephrectomy. The technique used for heterotopic renal transplantation was performed as previously described by Dar et. al.[13] Lewis rats were romized into 4 experimental groups: sensitization for 21 days followed by kidney transplant **(G4, sensitized transplant control; N = 6)**; sensitization for 21 days followed by desensitization with TACI-Ig treatment for 21 days (250 μg TACI-Ig (1mg/kg) in PBS pH 6.2, i.p. bolus injection 3 times per week for 3 weeks) followed by kidney transplant **(G5, sensitized + pre-transplant TACI-Ig; N = 6)**; sensitization for 21 days followed by kidney transplant with post-transplant TACI-Ig for 21 days **(G6, sensitized + post-transplant TACI-Ig; N = 7)**; sensitization for 21 days followed by desensitization with TACI-Ig treatment for 21 days followed by kidney transplant post-transplant TACI-Ig for 21 days **(G7, sensitized + pre- post-transplant TACI-Ig; N = 7)** (Table 1). TACI-Ig was used to block both APRIL BLyS. Animals were sacrificed on day 21 post-transplant tissues were collected for immediate utilization, were stored in 10% formalin for immunohistochemistry (IHC), were snap frozen in liquid nitrogen stored at -80°C for RT-PCR or Western blot, or were processed to single cells cryopreserved in liquid nitrogen. Plasma PBMC (peripheral blood mononuclear cell) were obtained at sacrifice by cardiac puncture into heparinized tubes.

**Table 1:** Kidney transplant experimental methodology.

Group	KTx (G4) (N = 6)	Pre-tx APRIL/BLyS blockade + KTx (G5) (N = 6)	KTx + post-tx APRIL/BLyS blockade (G6) (N = 7)	Pre-/post-tx APRIL/BLyS blockade + KTx (G7) (N = 7)
**CSA**	**+**	**+**	**+**	**+**
**0.5 mL heparinized BN blood for 21 days**	**+**	**+**	**+**	**+**
**250 μg APRIL/BLyS blockade given 3x weekly i.p. for 21 days pre-tx**	**-**	**+**	**-**	**+**
**250 μg APRIL/BLyS blockade given 3x weekly i.p. for 21 days post-tx**	**-**	**-**	**+**	**+**

Modified animal model of acute antibody mediated rejection with APRIL/BLyS blockade.

### Flow cytometry

Single cell suspensions of splenocytes, bone marrow, PBMC, mesenteric lymph nodes were prepared from fresh cells. After Ficoll purification, splenocytes, PBMC bone marrow underwent ACK lysis of red blood cells. After counting re-suspension in R10 (RPMI with 10% Fetal Calf Serum), 500,000 cells were added to cluster tubes stained. Cells from each tissue were stained for B lymphocyte subsets, marginal zone (MZ) B lymphocytes, T lymphocyte subsets, regulatory T cells (Tregs). The antibodies used for the B lymphocyte subsets were as follows: anti-IgD (clone MARD3, BioRad), anti-IgM (clone G53-238 BD Pharmingen), anti-CD38 (clone 14.27 BioLegend), anti-CD24 (clone ML5 BD Horizon), anti-CD45R (B220) (clone HIS24 eBioscience), anti-CD27 (clone LG.3A10 BD Horizon), anti-CD138 (clone B-A38 abcam). Antibodies used for the MZ B lymphocytes include anti-MZ B cell (clone HIS57 BD Pharmingen) anti-CD45RA (clone OX-33 BD Pharmingen). Antibodies used for the T lymphocyte subsets were as follows: anti-CD3 A647 (clone eBioG4.18 Invitrogen), anti-CD4 (clone W3/25 BioLegend), anti-CD8 (clone OX-8 BioLegend), anti-CCR4 (clone 205410 R&D Systems), anti-CCR6 (clone 876515 R&D Systems), anti-CCR10 (clone 248918 R&D Systems), anti-CD278 (clone C398.4A BioLegend), anti-CD3 (clone 1F4 BD Horizon), anti-CXCR5 (ab133706). Antibodies used for the Treg stain were anti-CD25 (clone OX-39 BioLegend), anti-CD4 (clone W3/25 BioLegend), anti-CD3 (clone 1F4 BD Horizon), anti-FoxP3 (clone 150D BioLegend). Flow cytometry was performed on a BD LSR II or BD LSR Fortessa at the UWCCC Flow Cytometry Laboratory data analyzed with FlowJo (TreeStar, Inc., Ashl, OR).

#### B lymphocyte subset gating

Cells were gated to remove non-singlets, then gated through a tight lymphocyte gate based on forward side scatter, were then visualized as IgD *versus* CD45R. From this gate, naïve B lymphocytes (IgD^+^CD45R^+^CD27^-^), non-switched B lymphocytes (IgD^+^CD45R^+^CD27^+^), transitional zone B lymphocytes (IgD^+^CD45R^+^CD38^+^CD24^+^) were defined. Switched B lymphocytes were defined as IgD^-^CD45R^+^IgM^-^CD27^+^. Memory B lymphocytes were defined as CD27^+^CD45R^+^ from the lymphocyte gate.

#### Plasma cell gating

Cells were gated to remove non-singlets, then through a large gate to ensure capture of the typically larger plasma cells, were subsequently visualized in an IgD *versus* CD45R gate. IgD^-^CD45R^-^ cells were then visualized as CD27 *versus* IgM. Plasma cells were defined as IgD^-^CD45R^-^CD27^+^IgM^-^CD138^+^. Normalized cell counts for plasma cells were calculated based on the large gate, rather than the lymphocyte gate as was used for other lymphocyte populations.

#### Marginal zone B lymphocyte gating

Cells were gated to remove non-singlets, then gated through a tight lymphocyte gate based on forward side scatter. From the lymphocyte gate, cells were visualized as MZ HIS57 *versus* CD45RA. MZ B lymphocytes were defined as HIS57^+^CD45RA^+^.

#### T lymphocyte subset gating

Cells were gated to remove non-singlets, then gated through a tight lymphocyte gate based on forward side scatter. Cells were then visualized as CD4 *versus* CD3 CD8 *versus* CD3. T lymphocytes were defined as CD4^+^CD3^+^ or CD8^+^CD3^+^. From each of these gates, T lymphocyte subsets were defined as CD4^+^CCR4^+^, CD4^+^CCR6^+^, CD4^+^CCR10^+^, CD4^+^CD278^+^, CD4^+^CXCR5^+^, CD8^+^CCR4^+^, CD8^+^CCR6^+^, CD8^+^CCR10^+^, CD8^+^CD278^+^, CD8^+^CXCR5^+^. T follicular helper (Tfh) cells were defined as CD4^+^CXCR5^+^CD278^+^, T follicular cells were defined as CD8^+^CXCR5^+^CD278^+^

#### Regulatory T cell gating

Cells were gated to remove non-singlets, then gated through a tight lymphocyte gate based on forward side scatter. Cells were visualized as CD4 *versus* CD3. CD4^+^CD3^+^ cells were defined as T lymphocytes then were further gated as CD25 *versus* FOXP3. Regulatory T cells (Tregs) were defined as CD4^+^CD3^+^CD25^+^FoxP3^+^.

#### Flow crossmatch

Flow crossmatch was performed using donor (Brown Norway) splenocytes freshly isolated from spleen, macerated through a 50 μm sieve, washed with R10 after red blood cell lysis with ACK. Cells were suspended, counted, 500,000 cells were aliquoted into cluster tubes for staining. Lewis rat serum from experimental time points was diluted 1:4 in R10 for a total volume of 50 μL, added to BN donor cells for 30 minutes at room temperature, washed, stained.[14, 15] Antibodies used include: anti-IgG1 (clone RG11/39.4 BD Bioscience), anti-IgG2a (clone RG7/1.30 BD Pharmingen), anti-IgG2b (clone RG7/11.1 BD Pharmingen), anti-IgG2c (clone A92-1 BD Pharmingen), anti-IgM (clone G53-238 BD Pharmingen), anti-CD3 (1F4 BD Horizon), anti-CD45R (B220) (clone HIS24 eBioscience). Cells were gated to remove non-singlets through a lymphocyte gate then a CD3^+^ or CD45R^+^ gate was used in order to perform T lymphocyte or B lymphocyte flow crossmatch, respectively. Mean fluorescence intensity (MFI) was determined for the population of interest.

### Histology

Kidneys were collected at time of euthanasia 1/3 of the spleen was preserved in 10% formalin for at least 24 hours, processed, paraffin embedded, cut into 5 μm sections. After deparaffinizing rehydrating, sections were stained with anti-PAX5 (Abcam, ab140341 polyclonal) or C4d (anti-C4d polyclonal antibody, American Research Products, Inc.) antibody overnight. The ImmPRESS HRP reagent ImmPACT DAB substrate were used to detect anti-PAX5 primary antibody as a brown pigment. Tissue sections were washed in distilled water, counter-stained with hematoxylin dehydrated through an ethanol series. Immunoperoxidase staining was done as previously described.[16, 17] Slides were imaged on a Nikon Eclipse E600 supplied with an Olympus DP70 camera. Automated quantification was performed using a custom macro written for ImageJ software (NIH, imagej.nih.gov/ij/). All hematoxylin-eosin C4d slides were reviewed by a transplant pathologist scored for peritubular capillaritis (ptc), glomerulitis (g), tubulitis (t), vasculitis (v), interstitial inflammation (i), mi (microcirculation inflammation) C4d staining, according to Banff 2013. Antibody mediated rejection (ABMR) acute cellular mediated rejection (ACMR) scores were calculated according to Banff 2013 guidelines.[18]

### ELISPOT

Single cell suspensions of splenocytes, bone marrow, mesenteric lymph nodes were prepared from fresh tissue with Ficoll purification. Cells were counted added to the plate (3654-WP-10 ManTech) previously coated with 10 μg/mL anti-IgG (315-005-046, JacksonImmuno Research Laboratories) or 10 μg/mL anti-IgM (315-005-049, JacksonImmuno Research Laboratories) in bicarbonate coating buffer, washed, then blocked. Cells were incubated overnight at 37°C in a 5% CO_2_ incubator. The following day cells were removed the plate was washed 5 times with PBS. Then 0.1 μg/mL biotinylated anti-IgG (315-065-046, JacksonImmuno Research Laboratories) or 0.1 μg/mL biotinylated anti-IgM (315-065-049, JacksonImmuno Research Laboratories) in PBS/0.1% Tween20 was added at 100 μL per well incubated for 2 hours at RT. After washing, ExtrAvidin-ALP (1:1000) (Sigma Aldrich, E2636) in PBS was added at 100 μL per well, incubated 1 hour at RT. After washing, 100 μL per well of substrate (3650–10 BCIP/NBT-plus MabTech) was added allowed to develop until distinct spots emerged. Color development was stopped by washing extensively in tap water. After drying, spots were counted by h using a dissecting microscope.

### RT-PCR

RT-PCR was performed using stard methodology.[14, 19] Snap frozen tissue was ground under liquid nitrogen to a powder, then RNA purified from the frozen powder using trizol (Invitrogen). RNA was cleaned up using RNA Easy (Qiagen) to remove any poor-quality RNA then reverse-transcribed by SuperScript IV (Invitrogen) according to manufacturer’s instructions subjected to RT-PCR using Taqman probes from Life Technologies using the Applied Biosciences Fast 7500 qPCR instrument. ΔΔCT was calculated, normalizing to ribosomal S26 using non-sensitized controls as the comparison for fold change.

### Statistics

Statistics were performed using the statistical packages that are part of Prism 7 for Mac OS X, v 7.0b. ANOVA, T-tests, chi-square were primarily used. *P* values of 0.05 or less were considered significant. Statistical calculations to determine power were determined prior to implementation of this experiment.

## Results

### Sensitized, APRIL/BLyS blockade treated rats

#### Plasma cells consistently decreased in APRIL/BLyS blockade treated animals

**Fig 2:**
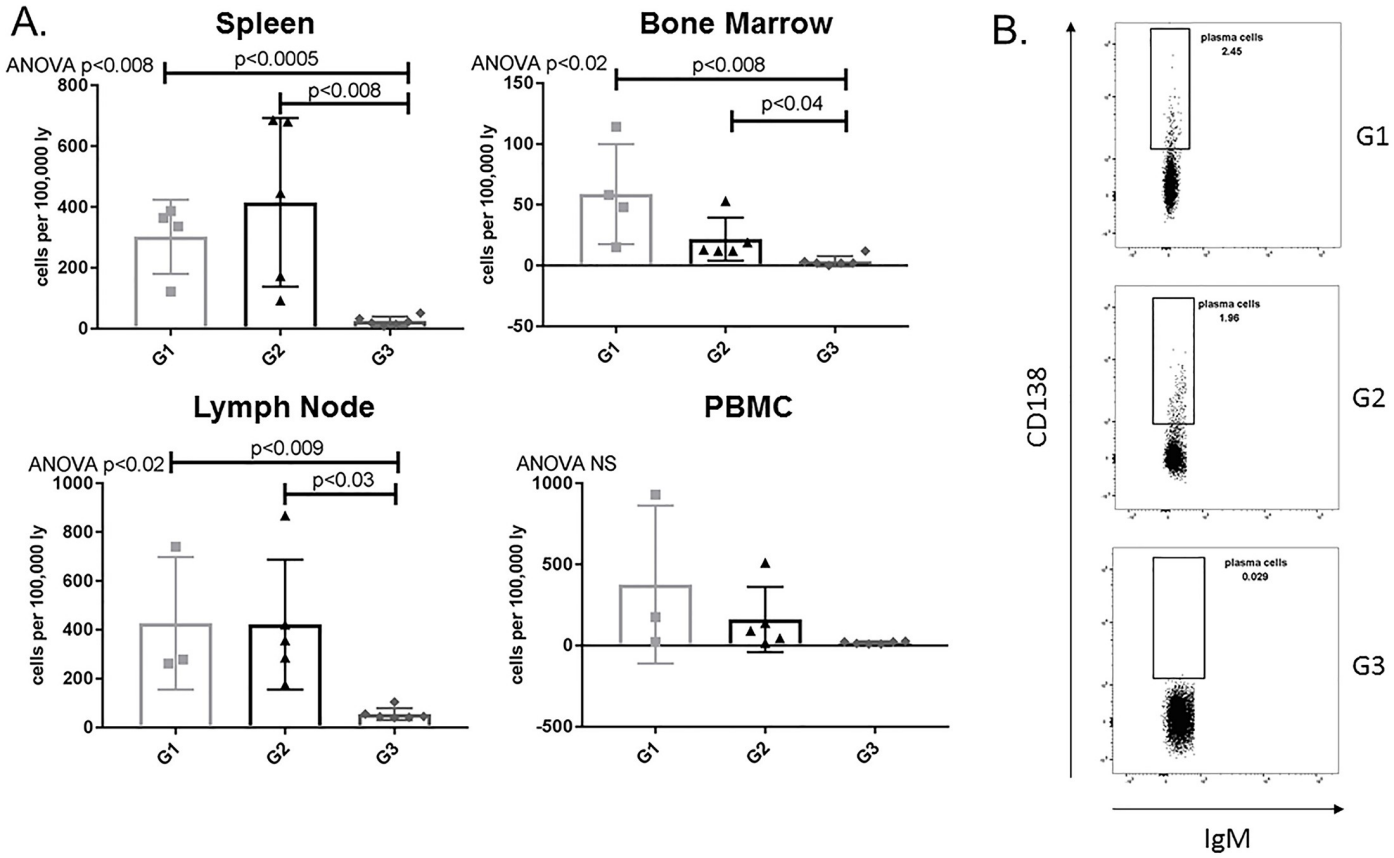
Plasma cells significantly decreased in APRIL/BLyS blockade treated group. Flow cytometry was used to assess plasma cell populations for each group. (A) Each graph shows number of cells per 100,000 lymphocytes. Plasma cells were defined as IgD^-^CD45R^-^CD27^+^IgM^-^CD138^+^. (B) Representative flow cytometry data of plasma cells in spleen. Number shown represents percentage of cells in gate.

In order to evaluate the efficacy of APRIL/BLyS blockade as a method to ultimately desensitize patients prevent ABMR, we began with the assessment of plasma cell presence functionality. Plasma cells (IgD^-^CD45R^-^CD27^+^IgM^-^CD138^+^ or IgD^-^CD45R^-^CD27^+^IgM^-^CD38^+^) were significantly depleted in APRIL/BLyS blockade treated rats in spleen, bone marrow, lymph nodes compared to both non-sensitized (p<0.009) sensitized untreated animals (p<0.04) (Fig 2).

#### APRIL/BLyS blockade treated animals demonstrated consistent depletion in IgG IgM secreting cells

**Fig 3:**
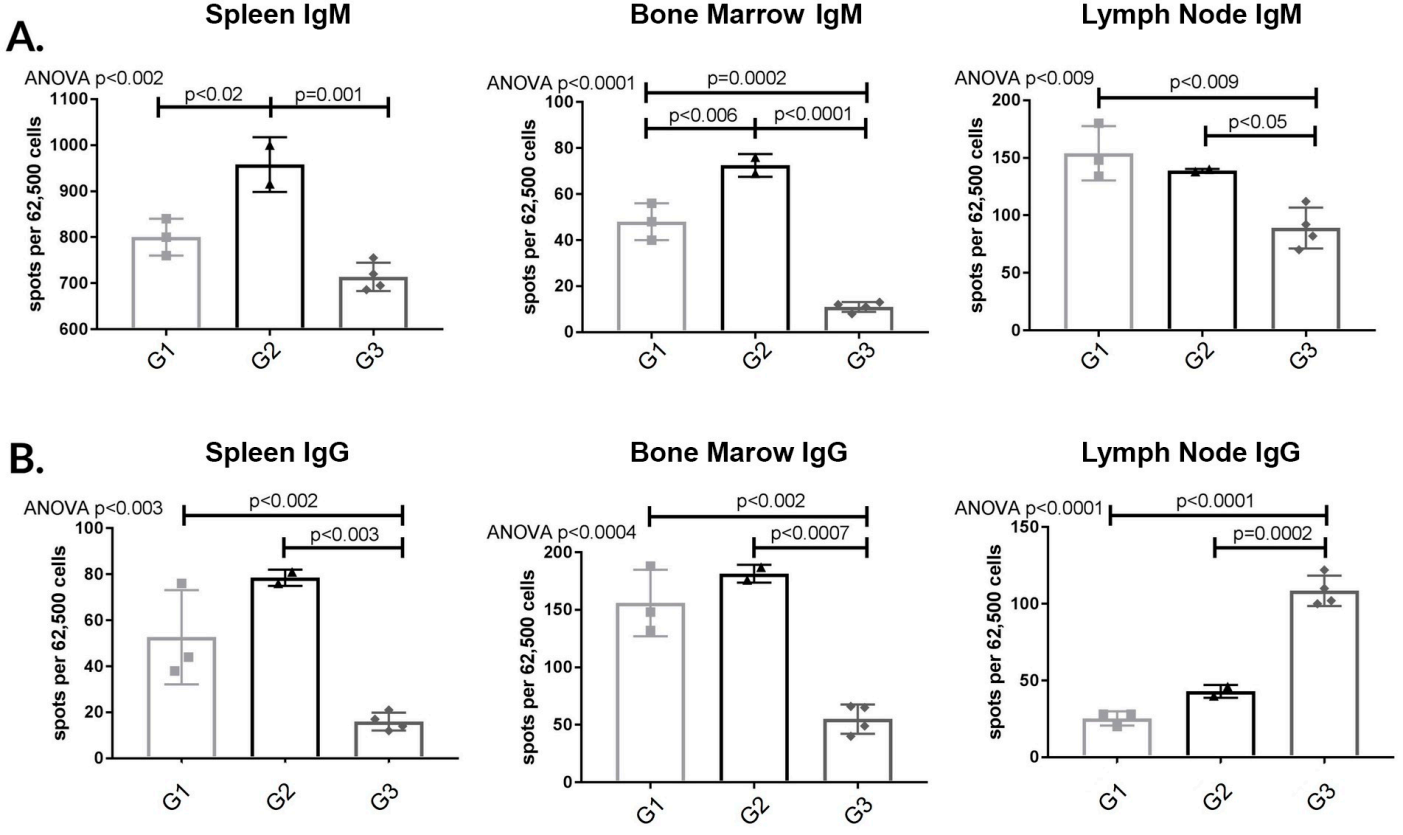
APRIL/BLyS blockade depleted IgM IgG secreting cells compared to non-sensitized sensitized untreated groups. Each graph shows number of spots per 62,500 lymphocytes. Spots represent antibody secreting cells. (A) IgM secreting cells. (B) IgG secreting cells. Together, decreased plasma cell antibody secreting cell populations add further support to the use of APRIL/BLyS blockade as a therapy to target potentially reduce antibody production.

After detecting a depletion of plasma cells in APRIL/BLyS blockade treated animals, we verified these results using ELISPOT in order to compare total antibody producing capabilities in APRIL/BLyS blockade treated animals to non-sensitized sensitized untreated animals. Overwhelmingly, APRIL/BLyS blockade resulted in a profound depletion of IgM IgG secreting cells across spleen bone marrow when compared to non-sensitized sensitized untreated animals (Fig 3). APRIL/BLyS blockade significantly decreased IgM secreting cells compared to non-sensitized in bone marrow lymph node (p<0.009) in all tissues compared to sensitized untreated animals (p<0.05) (Fig 3A). IgG secreting cells were significantly decreased in spleen bone marrow when comparing APRIL/BLyS blockade to both non-sensitized sensitized untreated animals (p<0.003) (Fig 3B). Of note, APRIL/BLyS blockade resulted in significantly increased IgG secreting cells in lymph nodes compared to both non-sensitized sensitized untreated animals (p<0.0002) (Fig 3B).

#### APRIL/BLyS blockade did not reduce donor specific antibodies (DSA)

**Fig 4:**
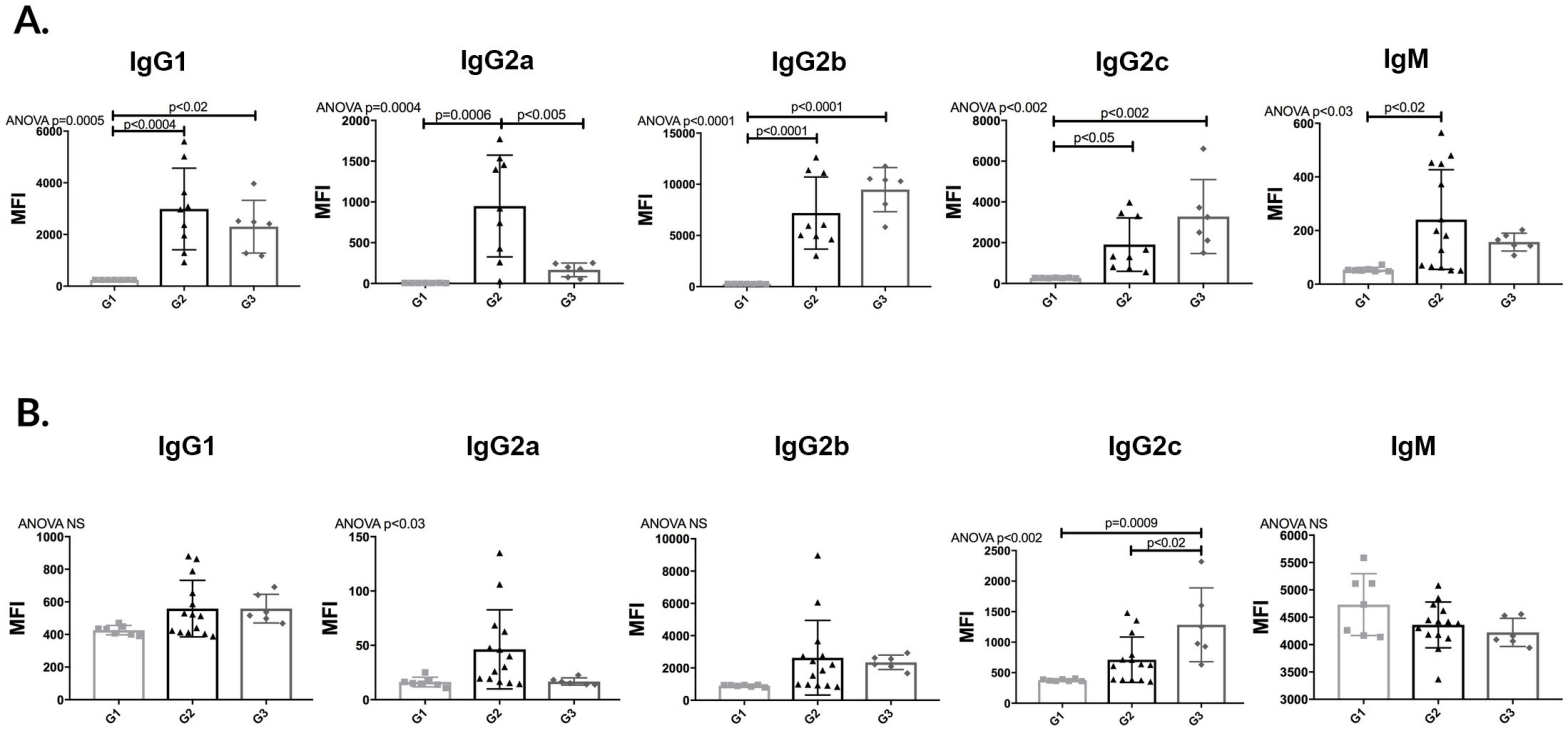
APRIL/BLyS blockade did not decrease donor specific antibody (DSA). (A) T (CD3^+^) lymphocyte flow cross match was performed as previously described. (B) B (CD45R^+^) lymphocyte flow cross match.

In order to determine the effect of APRIL/BLyS blockade on preformed alloantibody, Lewis rats were sensitized with BN blood for 3 weeks in order to allow for an alloantibody response. After 3 weeks, sensitized Lewis rats were treated with APRIL/BLyS blockade 3 times a week for 3 weeks were then compared to control groups non-sensitized sensitized untreated Lewis rats. B (CD45R^+^) T (CD3^+^) lymphocyte flow crossmatch was performed in order to measure DSA for IgG1, IgG2a, IgG2b, IgG2c, IgM. APRIL/BLyS blockade was found to significantly reduce CD3^+^IgG2a compared to sensitized untreated animals (p<0.005) (Fig 4A). As expected, in the T lymphocyte flow crossmatch, IgG1, IgG2a, IgG2b, IgG2c, IgM were significantly increased in G2 compared to G1. G3 animals were only found to have significantly decreased IgG2a compared to G2 animals in the T lymphocyte flow crossmatch (Fig 4A). In the B lymphocyte flow crossmatch, IgG2c was significantly increased in G3 compared to both G1 G2. No other significant differences in the B lymphocyte flow crossmatch were found between groups (Fig 4B). These findings suggest that despite a decrease in plasma cells as seen in flow cytometry antibody secreting cells demonstrated in ELISPOT data, desensitization with APRIL/BLyS blockade was unable to decrease DSA in this time period.

#### APRIL/BLyS blockade treated rats demonstrated a decrease in mature B lymphocyte populations

**Fig 5:**
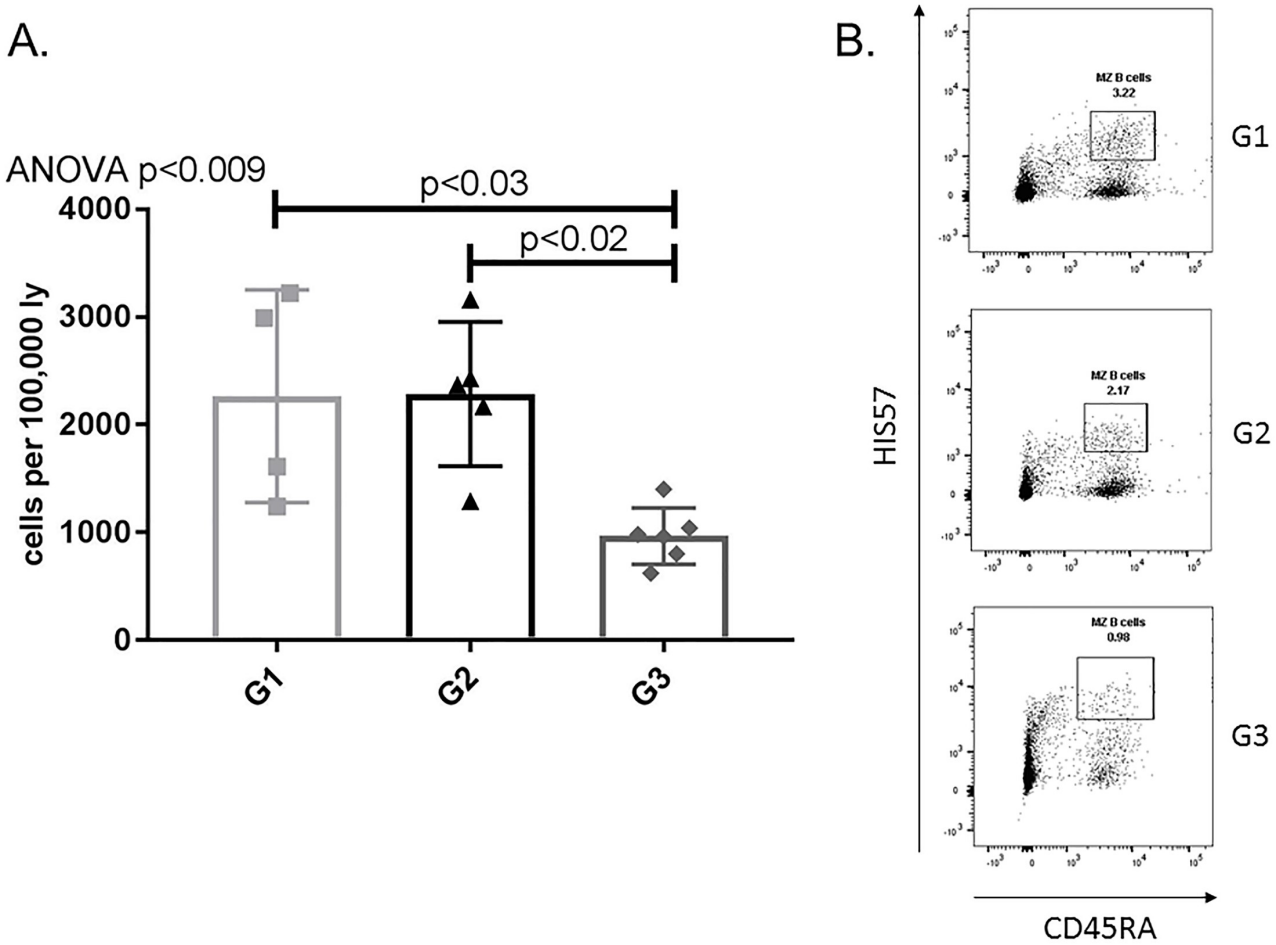
APRIL/BLyS blockade significantly reduced marginal zone B lymphocytes. Flow cytometry was used to assess marginal zone (MZ) B lymphocytes for each group. (A) Graph shows number of cells per 100,000 lymphocytes. MZ B lymphocytes were defined as HIS57^+^CD45RA^+^. (B) Representative flow cytometry data of MZ B lymphocytes in spleen. Number shown represents percentage of cells in gate.

**Fig 6:**
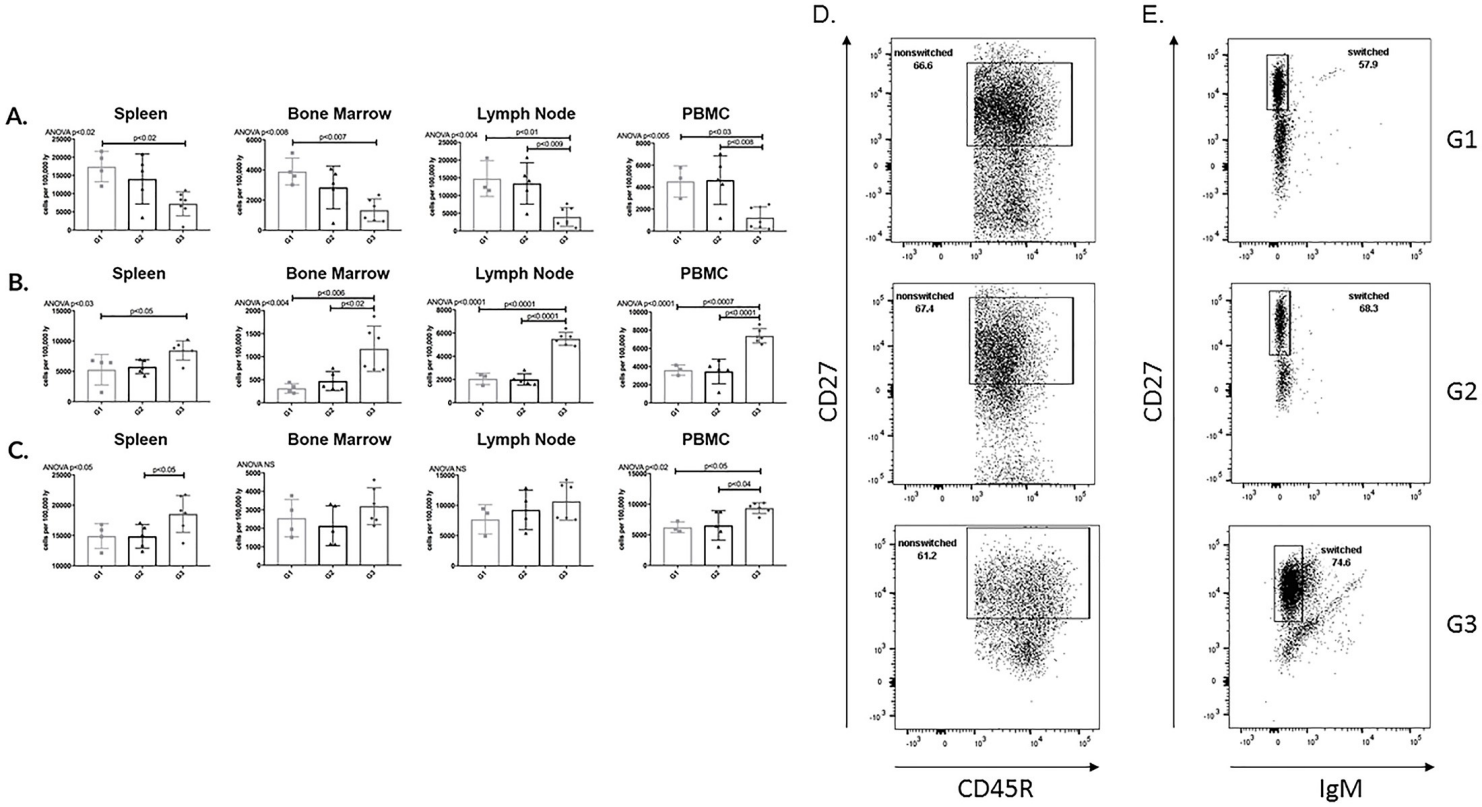
Non-switched B lymphocytes significantly reduced with APRIL/BLyS blockade. Flow cytometry was used to assess mature B lymphocyte populations for each group. Each graph shows number of cells per 100,000 lymphocytes. (A) Non-switched B lymphocytes were defined as IgD^+^CD45R^+^CD27^+^. (B) Switched B lymphocytes were defined as IgD^-^CD45R^+^IgM^-^CD27^+^. (C) Memory B cells were defined as CD27^+^CD45R^+^. Representation flow cytometry data of (D) non-switched (E) switched B lymphocytes in spleen. Number shown represents percentage of cells in gate.

After evaluating plasma cells, we examined the effect of APRIL/BLyS blockade on other B lymphocyte populations. Sensitized rats treated with APRIL/BLyS blockade consistently demonstrated a decrease in mature B lymphocyte subsets Sensitized rats treated with APRIL/BLyS blockade had a significant decrease in spleen MZ (HIS57^+^CD45RA^+^) populations compared to non-sensitized sensitized untreated rats (p<0.03) (Fig 5). APRIL/BLyS blockade treated rats also had an overall decrease in non-switched B lymphocyte populations (IgD^+^CD45R^+^CD27^+^), which was found to be significantly lower in all tissues compared to non-sensitized (p<0.03) in lymph nodes PBMC compared to sensitized untreated animals (p<0.009) (Fig 6A). Switched B lymphocyte (IgD^-^CD45R^+^IgM^-^CD27^+^) populations were found to be elevated in APRIL/BLyS blockade treated animals compared to both non-sensitized in all tissues (p<0.05) sensitized untreated animals in bone marrow, lymph node PBMC (p<0.02) (Fig 6B). APRIL/BLyS blockade resulted in an increase in spleen PBMC memory B cells (CD27^+^CD45R^+^) compared to sensitized untreated animals (p<0.05) (Fig 6C).

#### APRIL/BLyS blockade reduced transitional zone but not naïve B lymphocytes

**Fig 7:**
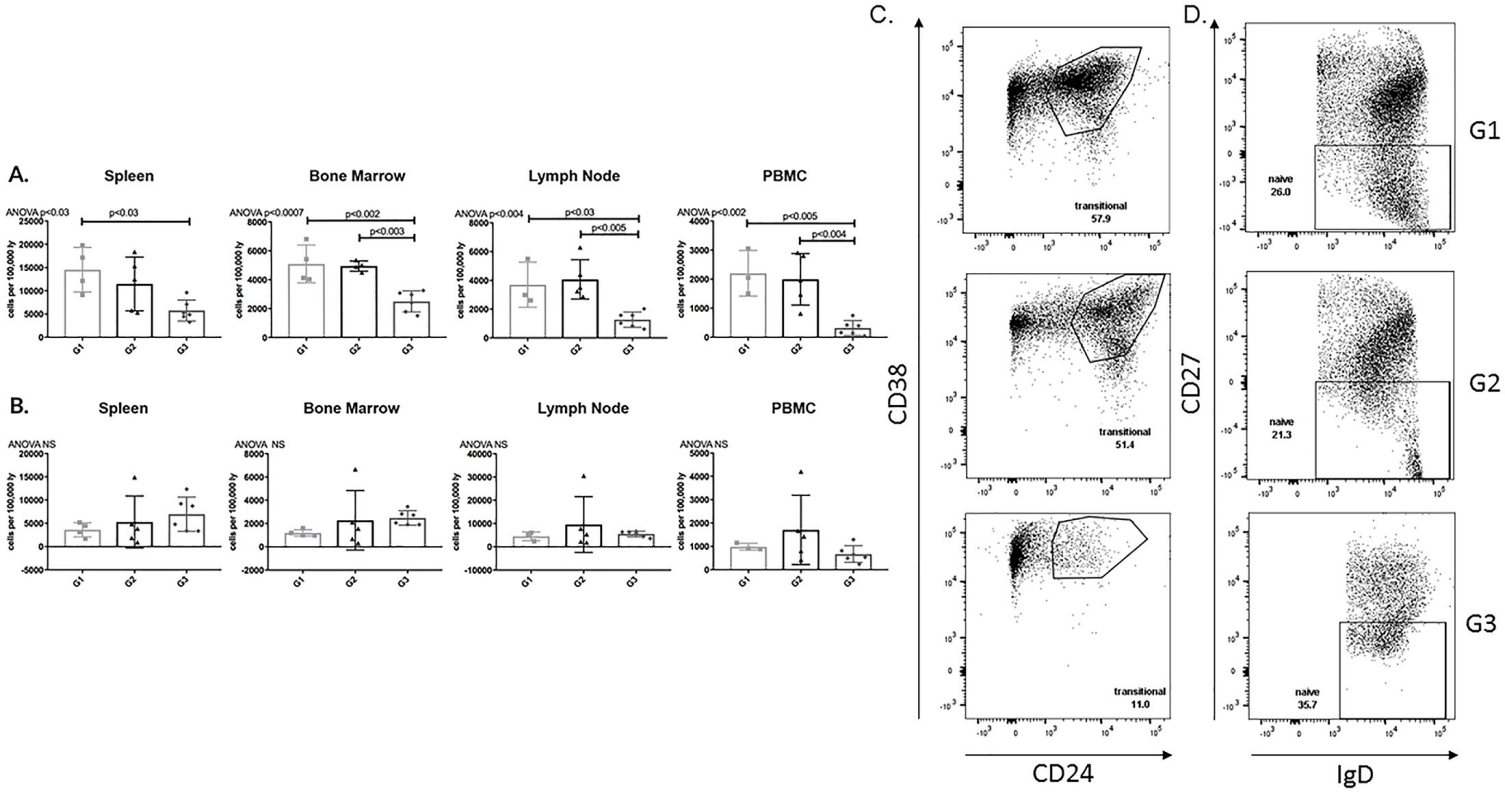
Transitional Zone (TZ) B cells significantly decreased with APRIL/BLyS blockade compared to sensitized untreated group. Flow cytometry was used to assess immature B lymphocyte populations for each group. Each graph shows number of cells per 100,000 lymphocytes. (A) Transitional zone (TZ) B cells were defined as IgD^+^CD45R^+^CD38^+^CD24^+^. (B) Naïve B cells were defined as IgD^+^CD45R^+^CD27^-^. Representative flow cytometry data of (C) TZ B cells (D) naïve B cells in spleen. Number shown represents percentage of cells in gate.

APRIL/BLyS blockade treated rats demonstrated a significantly smaller population of transitional zone (TZ) lymphocytes (CD24^+^CD38^+^) in all tissues compared to non-sensitized animals (p<0.03). APRIL/BLyS blockade also resulted in a significantly smaller population of TZ in bone marrow, lymph nodes, PBMC compared to sensitized untreated animals (p<0.005). Naïve B lymphocytes (IgD^+^CD27^-^) were preserved following desensitization with APRIL/BLyS blockade (Fig 7).

These findings indicate that in addition to depleting plasma cells, terminally differentiated B lymphocytes responsible for alloantibody production, APRIL/BLyS blockade also altered the composition of mature immature B lymphocyte populations. The depletion of B lymphocyte populations in addition to plasma cells is critical due to the role that B lymphocytes play as antigen presenting cells.[20, 21] After establishing the effect of APRIL/BLyS blockade on B lymphocyte populations, the second aim of this study was to determine if these results could be duplicated in the context of an ABMR kidney transplant model.

### Sensitized transplanted rats with differential timing of APRIL/BLyS blockade treatment

Lewis rats underwent kidney transplantation from a donor BN with bilateral nephrectomy. All animals were sensitized for 21 days received a course of CSA beginning on postoperative day 0. As previously stated, the groups were as follows: (G4) sensitization followed by kidney transplant **(sensitized transplant control)**; (G5) sensitization followed by desensitization with TACI-Ig (APRIL/BLyS blockade) prior to kidney transplant **(sensitized + pre-transplant TACI-Ig)**; (G6) sensitization followed by kidney transplant with post-transplant TACI-Ig **(sensitized + post-transplant TACI-Ig)**; (4G7 sensitization followed by desensitization TACI-Ig treatment followed by kidney transplant post-transplant TACI-Ig **(sensitized + pre- post-transplant TACI-Ig)** (Table 1).

#### Animals receiving APRIL/BLyS blockade post-transplant demonstrated significantly less antibody mediated rejection

Twenty-one days after kidney transplant, animals were sacrificed their tissues collected in order to evaluate the effect on APRIL/BLyS blockade on B lymphocyte populations the presence of ABMR in transplanted kidneys. All frozen specimen C4d slides were reviewed by a transplant pathologist (blinded) scored for peritubular capillaritis (ptc), glomerulitis (g), tubulitis (t), vasculitis (v), interstitial inflammation (i), mi (microcirculation inflammation) C4d according to Banff 2013. Animals in G6 did not develop any moderate ABMR (≥2) with only 1 animal developing mild ACMR. In support of this finding, no animal in G6 developed moderate or severe C4d deposition (≥2) or moderate mi (≥2, g + ptc) (Table 2).

**Table 2:** Animals treated with post-transplant APRIL/BLyS blockade did not develop antibody mediated rejection.

Group	C4d*	t*	v	i*	g*	ptc	mi	ABMR Banff (n)^▲^	ACMR Banff (n)
**Tx control (G4)**	0.0±0.0^1^	1.0±0.9^2^	0±0.0	1.3±1.0^3^	0.3±0.5	0.7±1.2	1.0±1.7	16.6% (1)	33.3% (2)
**Pre-tx APRIL/BLyS blockade (G5)**	1.5±1.2^1^	0.0±0.0^2^	0.0±0.0	0.0±0.0^3^	0.8±0.8^4^	0.8±0.4	1.7±1.0	83.3% (5)	0% (0)
**Post-tx APRIL/BLyS blockade (G6)**	0.4±0.5	0.1±0.4^2^	0.0±0.0	0.3±0.8	0.0±0.0^4^	0.1±0.4	0.1±0.4	0% (0)	14.3% (1)
**Pre-/post-tx APRIL/BLyS blockade (G7)**	1.1±0.7	0.6±0.5	0.0±0.0	0.6±0.5	0.0±0.0^4^	0.7±0.5	0.7±0.5	57.1% (5)	0% (0)

Kidney tissue was evaluated for Banff criteria at 21 days post-transplant. Groups listed in far left column as previously described.

^1^p<0.01 for C4d in Tx control compared with pre-tx APRIL/BLyS blockade

^2^p<0.05 for t in Tx control compared with pre-tx APRIL/BLyS blockade post-tx APRIL/BLyS blockade

^3^p<0.02 for i in Tx control compared with pre-tx APRIL/BLyS blockade

^4^p<0.02 for g in pre-tx APRIL/BLyS blockade compared with post-tx APRIL/BLyS blockade pre-/post-tx APRIL/BLyS blockade

*ANOVA p<0.02

^▲^p<0.004

Conversely, a majority of animals in G5 G7 developed of ABMR. Two-thirds animals in G5 demonstrated severe C4d deposition moderate mi. 83.3% of these animals developed ABMR. Findings similar to these were found in G7 animals. In this group, 57.1% developed ABMR with 57.1% 29% demonstrating moderate or severe C4d deposition, respectively. Moderate mi was seen in 71%.

**Fig 8:**
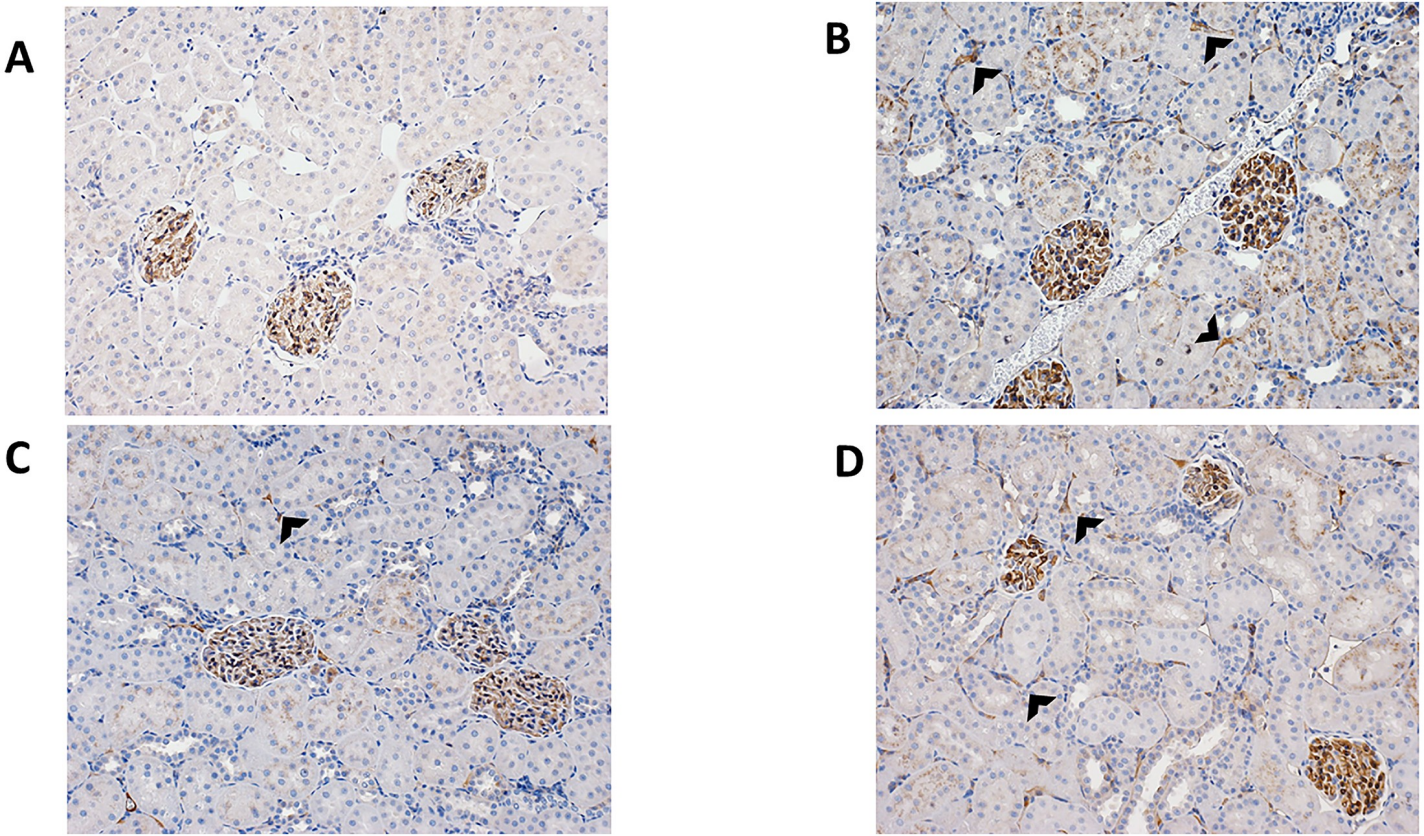
Post-transplant treated APRIL/BLyS blockade animals demonstrated less C4d deposition on biopsy. Representative histological depictions of (A) sensitized transplant control group (G4) with grade 0 C4d deposition; (B) pre-transplant APRIL/BLyS blockade group (G5) with grade 3 C4d deposition; (C) post-transplant APRIL/BLyS blockade group (G6) with grade 1 C4d deposition; (D) both pre- post-transplant APRIL/BLyS blockade group (G7) with grade 2 C4d deposition. Arrowheads indicate C4d deposition.

Of note, the control transplant group (G4) did not develop any moderate ABMR ≥2 did not demonstrate any C4d deposition (Fig 8).

These vastly different findings between groups indicate that timing of APRIL/BLyS blockade has a significant effect on the composition of B T lymphocytes present following transplant, which in turn impacts the development of ABMR or ACMR. Additionally, lack of concurrent T lymphocyte depletion via CSA could also have played a role in ABMR development in animals receiving pre-transplant APRIL/BLyS blockade (G5).

#### APRIL/BLyS blockade pre-transplant both pre- post-transplant did not result in acute cellular mediated rejection

All animals that underwent transplant received daily CSA in order to minimize the development of ACMR. Despite this 33.3% of control animals (G4) developed ACMR IA 14.3% of animals in G6 developed ACMR IA. No animals in the groups G5 or G7 developed ACMR (Table 2).

#### APRIL/BLyS blockade post-transplant resulted in a significant decrease in antibody secreting cells compared to pre-transplant treatment

**Fig 9:**
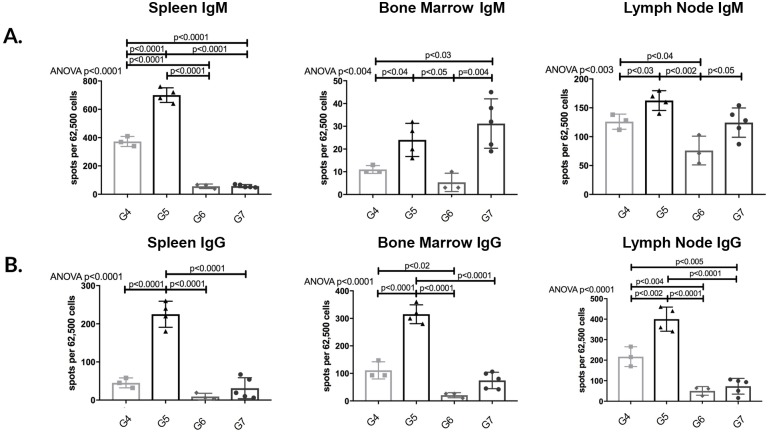
Post-transplant APRIL/BLyS blockade resulted in consistent depletion of IgM IgG secreting cells. ELISPOT was used to quantify antibody secreting cells including plasmablasts plasma cells. Each graph shows number of spots per 62,500 lymphocytes. Spots represent antibody secreting cells. (A) IgM (B) IgG secreting cells.

**Fig 10:**
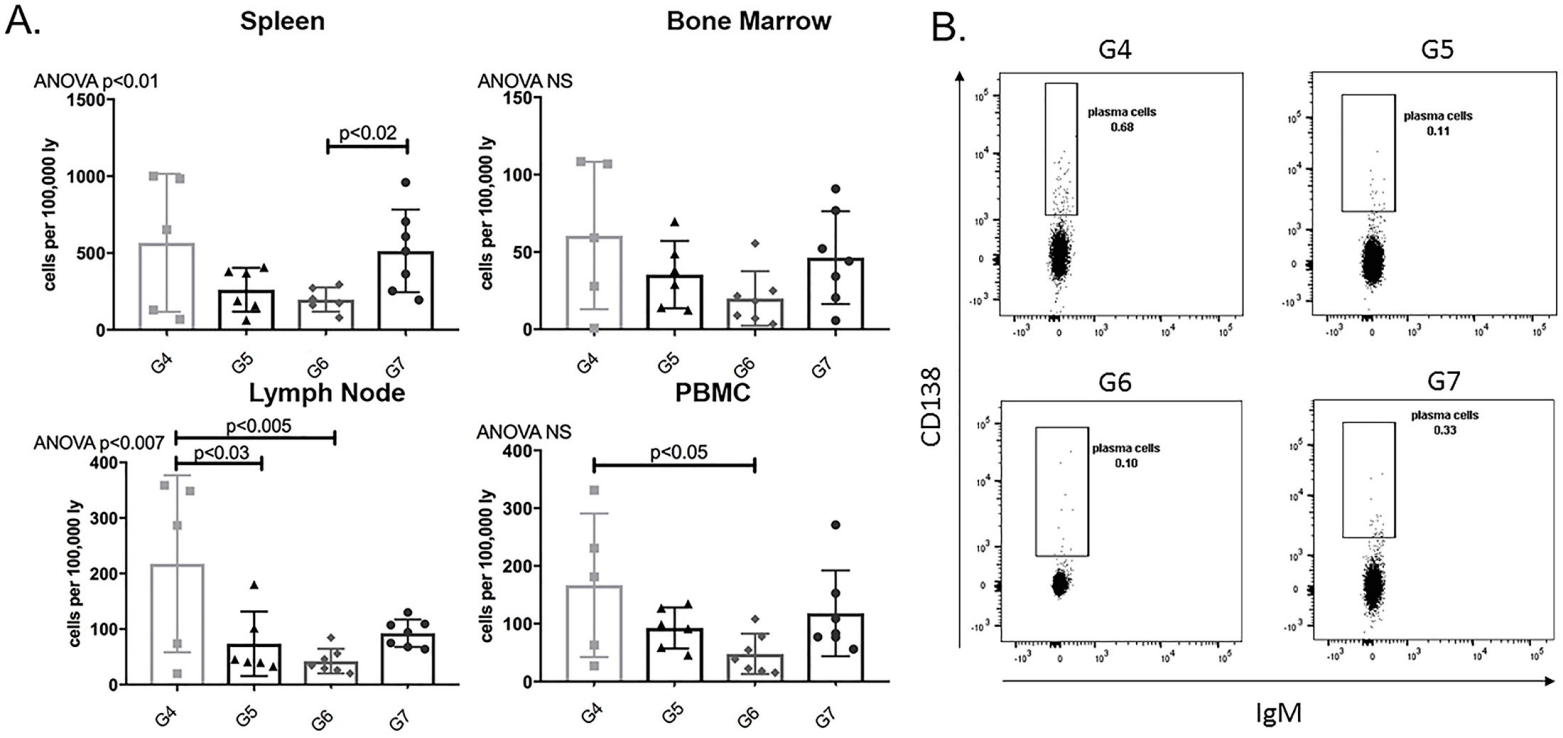
Plasma cells were decreased in post-transplant APRIL/BLyS blockade treated group in lymph node PBMC compared to transplant control. Flow cytometry was used to assess plasma cell populations for each group. (A) Each graph shows number of cells per 100,000 lymphocytes. Plasma cells were defined as IgD^-^CD45R^-^CD27^+^IgM^-^CD138^+^. (B) Representative flow cytometry data of plasma cells in lymph node. Number shown represents percentage of cells in gate.

We used ELISPOT assays in order to determine if the lack of ABMR development correlated with a change in IgG IgM secreting cells. Overall, G6 consistently depleted both IgG IgM secreting cells compared to all groups including transplant control (G4). Consistent with the significant levels of ABMR found on biopsy, G5 demonstrated a substantial increase in IgM IgG secreting cells in all tissues compared to G4 G6 (p<0.04) (Fig 9). Furthermore, G5 resulted in greater IgM IgG secreting cell production even compared to G7 in all tissues except IgM secreting cell in lymph node (p<0.05) (Fig 9). To reinforce our ELISPOT findings, plasma cell populations were assessed via flow cytometry. G6 showed decreased plasma cells in lymph node PBMC compared to G4 (p<0.05) (Fig 10). These findings offer further support to the idea that timing of lymphocyte depletion relative to transplant plays a significant role in remodeling of the B lymphocyte compartment.

#### No change in DSA in APRIL/BLyS blockade treated transplanted rats

**Fig 11:**
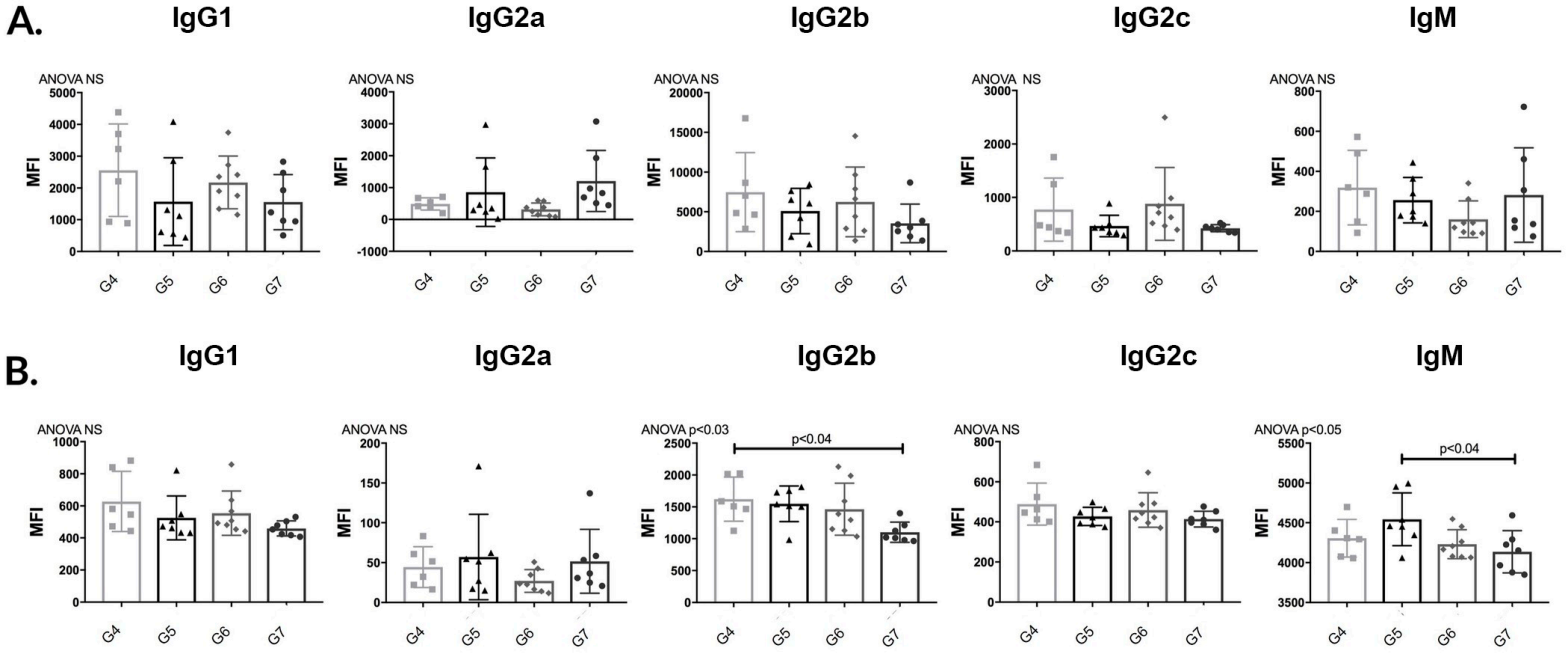
APRIL/BLyS blockade did not decrease donor specific antibody in sensitized transplant groups. (A) T (CD3^+^) (B) B (CD45^+^) lymphocyte flow cross match was performed as previously described.

B (CD45R^+^) T (CD3^+^) lymphocyte flow crossmatch was performed in order to determine if the timing of APRIL/BLyS blockade relative to transplant impacted DSA production. MFI for IgG1, IgG2a, IgG2b, IgG2c, IgM were measured in both B T lymphocyte flow crossmatch. Despite the fact that less ABMR was seen on biopsy fewer antibody secreting cells were noted on ELISPOT, G6 did not show a reduction in DSA compared to groups that did develop significant ABMR (G5 G7). B lymphocyte flow crossmatch only showed a decrease in IgG2b in G7 compared to G4 a decrease in IgM in G7 compared to G5 (Fig 11). More investigation will be required in order to determine why groups with less ABMR on biopsy did not overwhelming show less DSA production.

#### Post-transplant APRIL/BLyS blockade demonstrated superior depletion of B lymphocytes from splenic germinal centers compared to pre-transplant treated animals

**Fig 12:**
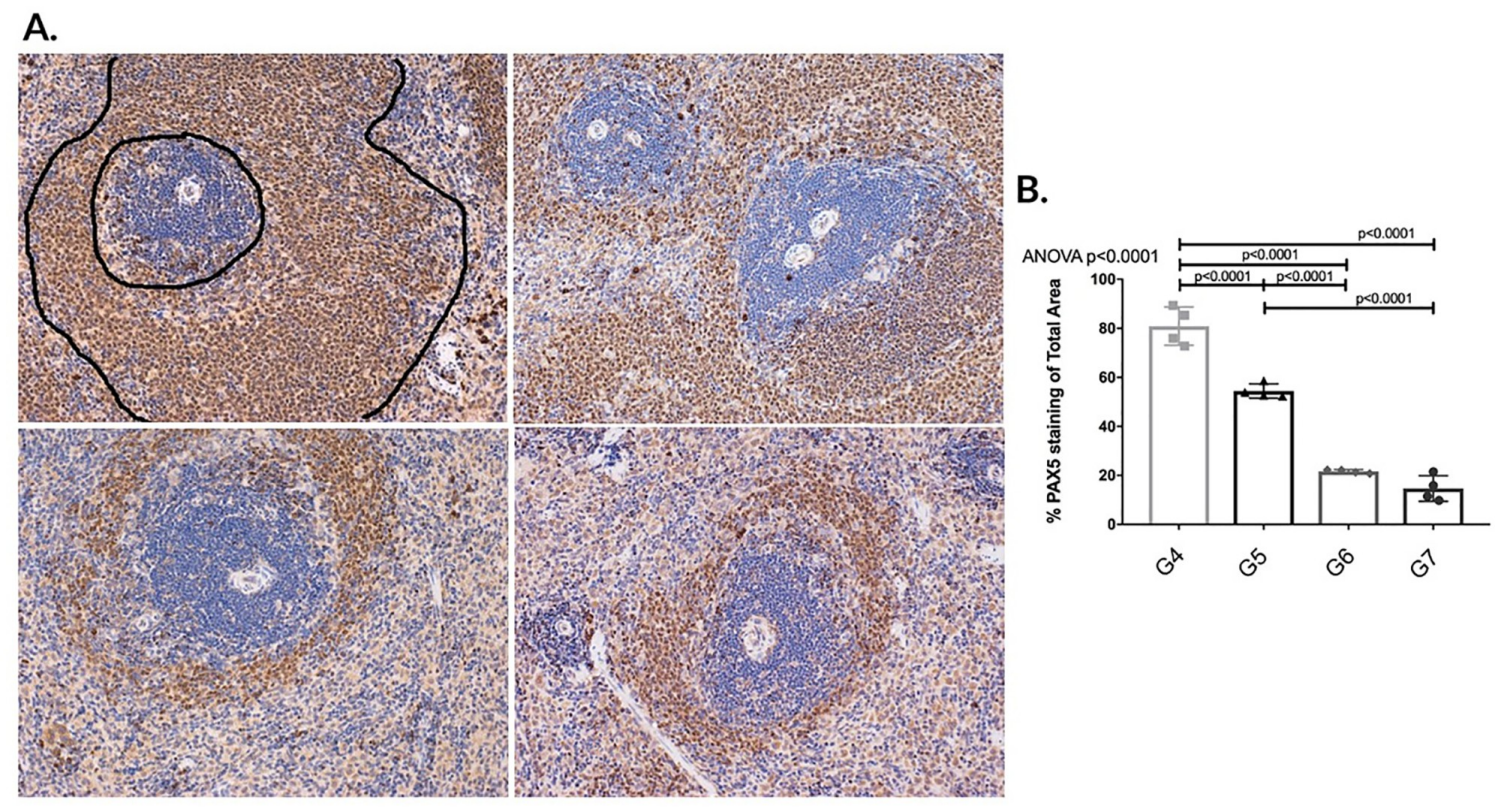
Post-transplant APRIL/BLyS blockade significantly depleted B lymphocytes from splenic germinal centers compared to pre-transplant treatment. Top row (left to right): sensitized transplant control group (G4); sensitized pre-transplant APRIL/BLyS blockade group (G5). Bottom row (left to right): sensitized post-transplant APRIL/BLyS blockade group (G6); sensitized both pre- post-transplant APRIL/BLyS blockade (G7). (A) Sensitized transplant control animals (G4) demonstrated normal architecture of germinal centers (outlined). (B) Densitometry using anti-PAX5 antibody to detect B lymphocytes in splenic germinal centers. Graph depicts percentage of total area of spleen staining for anti-PAX5.

**Fig 13:**
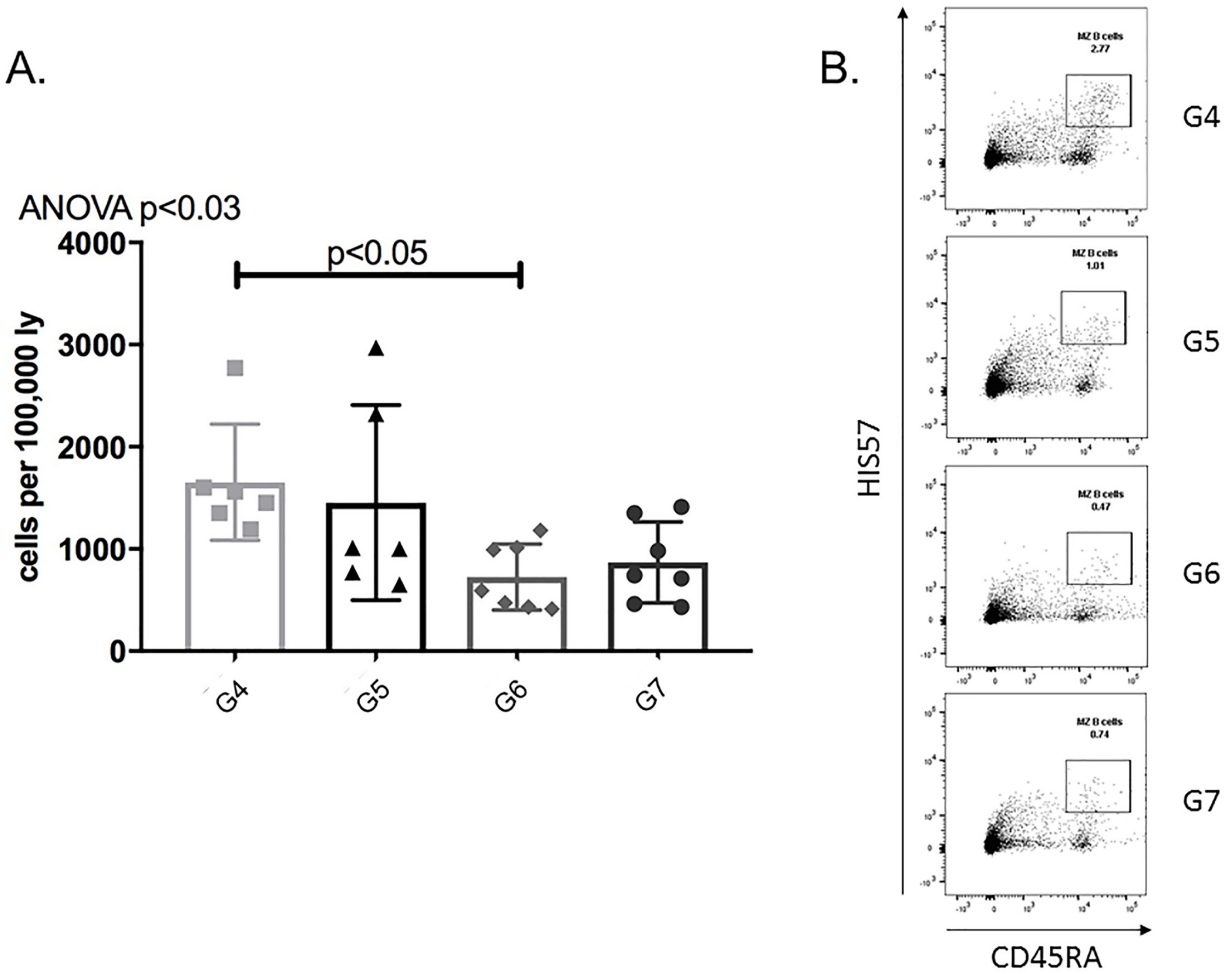
Splenic marginal zone B lymphocytes decreased in post-transplant APRIL/BLyS blockade compared to transplant control. Flow cytometry was used to assess MZ B lymphocytes for each group. (A) Each graph shows number of cells per 100,000 lymphocytes. MZ B lymphocytes were defined as HIS57^+^CD45RA^+^. (B) Representative flow cytometry data of spleen MZ B lymphocytes. Number shown represents percentage of cells in gate.

B lymphocyte populations in splenic germinal centers were characterized via flow cytometry IHC using PAX5 antibody. Animals in G4 demonstrated normal architecture of germinal centers (outlined) G5 displayed significant B lymphocyte depletion compared to G4. Animals in G6 G7 demonstrated a significant disruption of B lymphocytes found in splenic germinal centers compared to animals in G4 G5 as evidenced through overall decreased PAX5 staining (p<0.0001) (Fig 12B). To further support this finding, splenic MZ B lymphocytes were also found to be significantly decreased in G6 compared to G4 (p<0.05) (Fig 13). These findings of disrupted depleted germinal centers in addition to significantly decreased splenic IgM IgG secreting cells suggest that post-transplant APRIL/BLyS blockade in sensitized animals results in not only an overall decrease of B lymphocyte populations but also fewer active IgM IgG secreting cells. Ultimately, these findings are further upheld by the fact that significantly less ABMR was seen in the G6 compared to G5.

#### Memory B cells decreased in post-transplant APRIL/BLyS blockade treated rats

**Fig 14:**
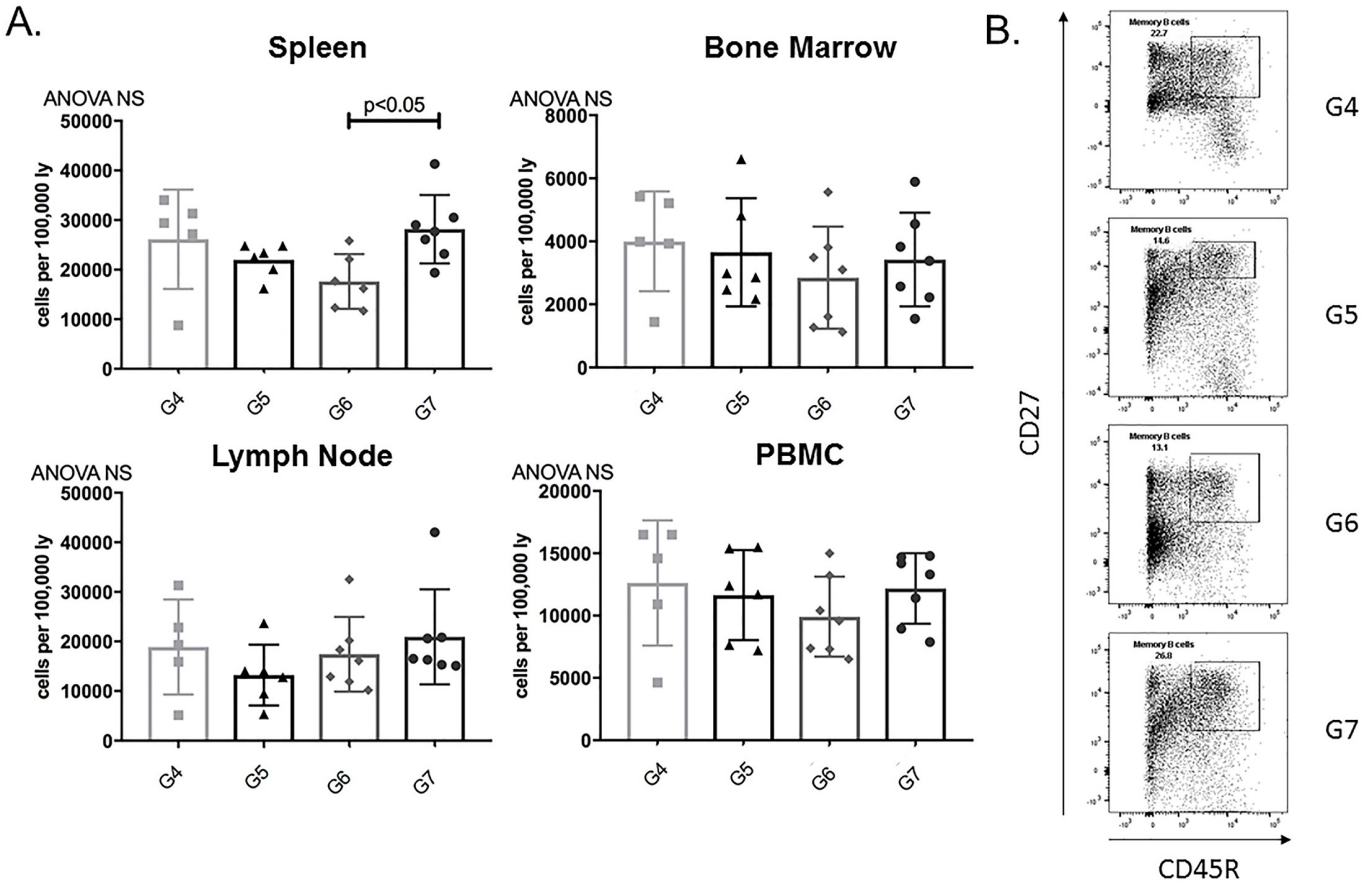
Splenic memory B cells significantly decreased in post-transplant APRIL/BLyS blockade compared to both pre- post-transplant APRIL/BLyS blockade. Flow cytometry was used to assess mature B lymphocyte populations for each group. (A) Each graph shows number of cells per 100,000 lymphocytes. Memory B cells were defined as CD27^+^CD45R^+^. (B) Representative flow cytometry data of spleen memory B lymphocytes. Number shown represents percentage of cells in gate.

In addition to changes seen in alloantibody production the subsequent development of ABMR, we used flow cytometry in order to determine if these changes were accompanied by changes in B lymphocyte populations. Memory B cells in spleen were significantly decreased in G6 animals compared to G7 (p<0.05) (Fig 14). Other mature B lymphocyte populations including non-switched switched B lymphocytes were evaluated for changes but none were noted between groups (S1 Fig).

Immature B lymphocyte subsets, known to be more sensitive specifically to BLyS depletion, were also evaluated via flow cytometry.[22] No differences were noted in naïve or TZ B lymphocyte populations between groups receiving APRIL/BLyS blockade or transplant control groups (S2 Fig).

#### Post-transplant APRIL/BLyS blockade significantly increased TFH (T follicular helper) lymphocytes

**Fig 15:**
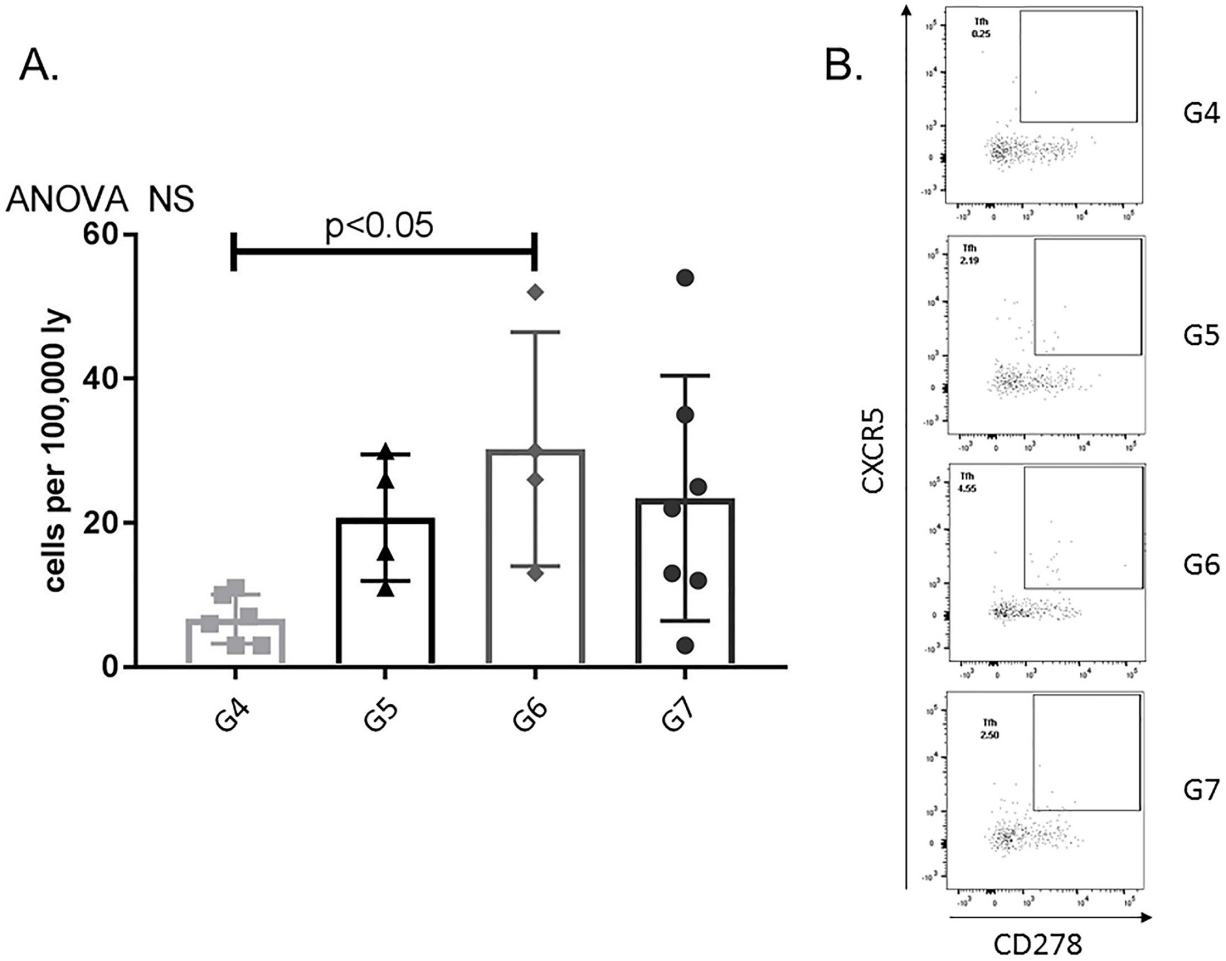
T follicular helper (TFH) lymphocytes significantly increased in post-transplant APRIL/BLyS blockade treated group compared to transplant control. Flow cytometry was used to assess TFH lymphocyte populations for each group. (A) Graph shows number of cells per 100,000 lymphocytes. TFH lymphocytes were defined as CD4^+^CXCR5^+^CD278^+^. (B) Representative flow cytometry data of bone marrow TFH lymphocytes. Number shown represents percentage of cells in gate.

Due to the fact that B lymphocyte inhibition has been shown to alter the differentiation activation of T lymphocytes, flow cytometry was used to assess for any changes in this population. G6 was found to have significantly increased TFH lymphocytes in the bone marrow compared to transplant control (p<0.05) (Fig 15).

**Fig 16:**
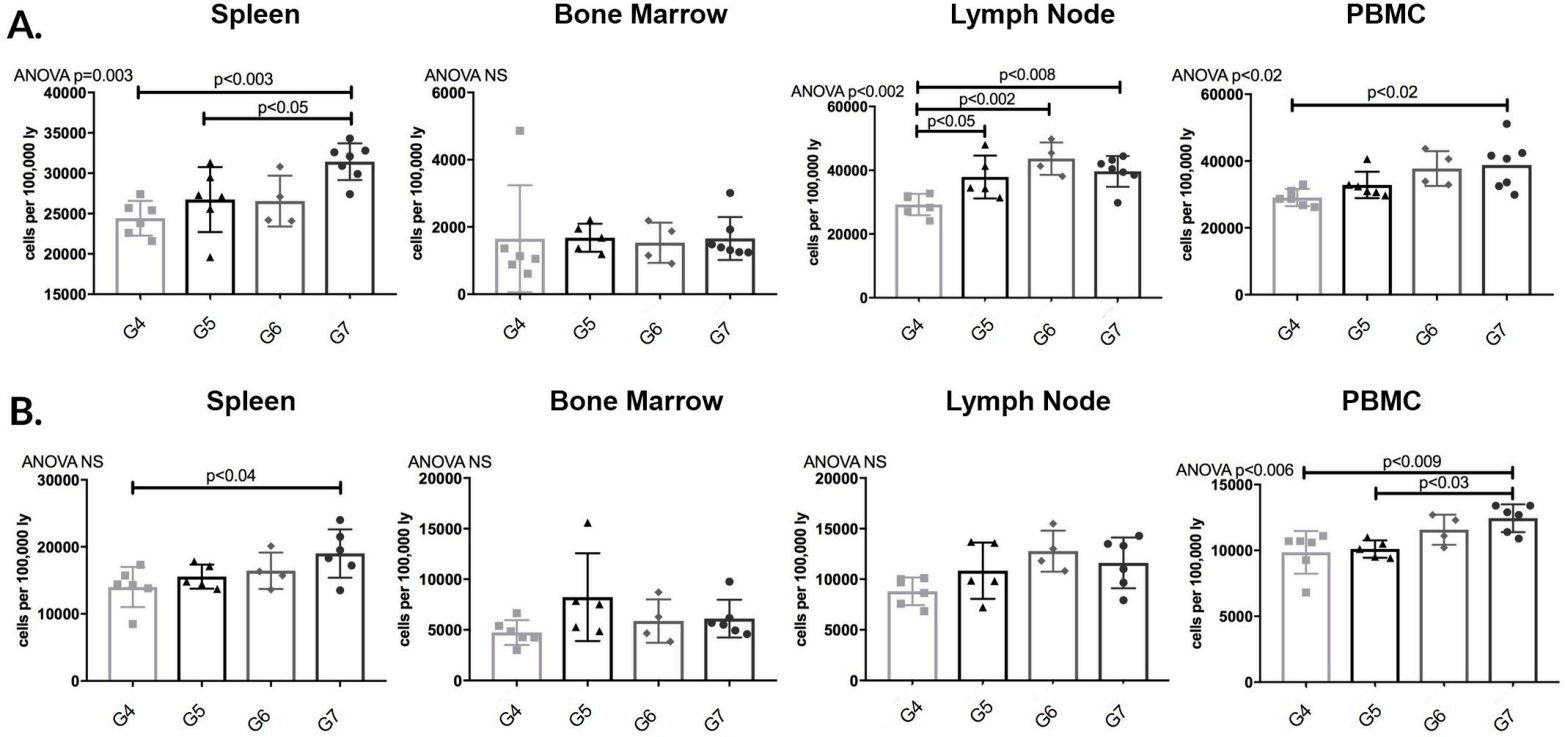
CD3^+^CD4^+^ CD3^+^CD8^+^ T lymphocytes increased in both pre- post-transplant APRIL/BLyS blockade compared to transplant control. Flow cytometry was used to assess T lymphocyte populations for each group. Each graph shows number of cells per 100,000 lymphocytes. (A) T lymphocytes were further defined as CD3^+^CD4^+^. (B) T lymphocytes defined as CD3^+^CD8^+^.

Lymph node CD3^+^CD4^+^ T lymphocytes were significantly increased in all groups compared to G4 (p<0.05). Animals in G7 were noted to have a significant increase in spleen PBMC CD3^+^CD4^+^ T lymphocytes compared to G4; this population was also significantly increased compared to G5 animals in spleen (p<0.05) (Fig 16A).

Animals in G7 demonstrated a significant increase in spleen PBMC CD3^+^CD8^+^ compared to G4 (p<0.04) (Fig 16B). This increase seen in CD3^+^CD4^+^ CD3^+^CD8^+^ T lymphocytes specifically in G7 animals could potentially be the result of a depletion of regulatory B lymphocytes that normally function to inhibit T lymphocyte proliferation.

#### APRIL/BLyS blockade post-transplant resulted in more IL-10 production compared to pre- post-transplant blockade

**Fig 17:**
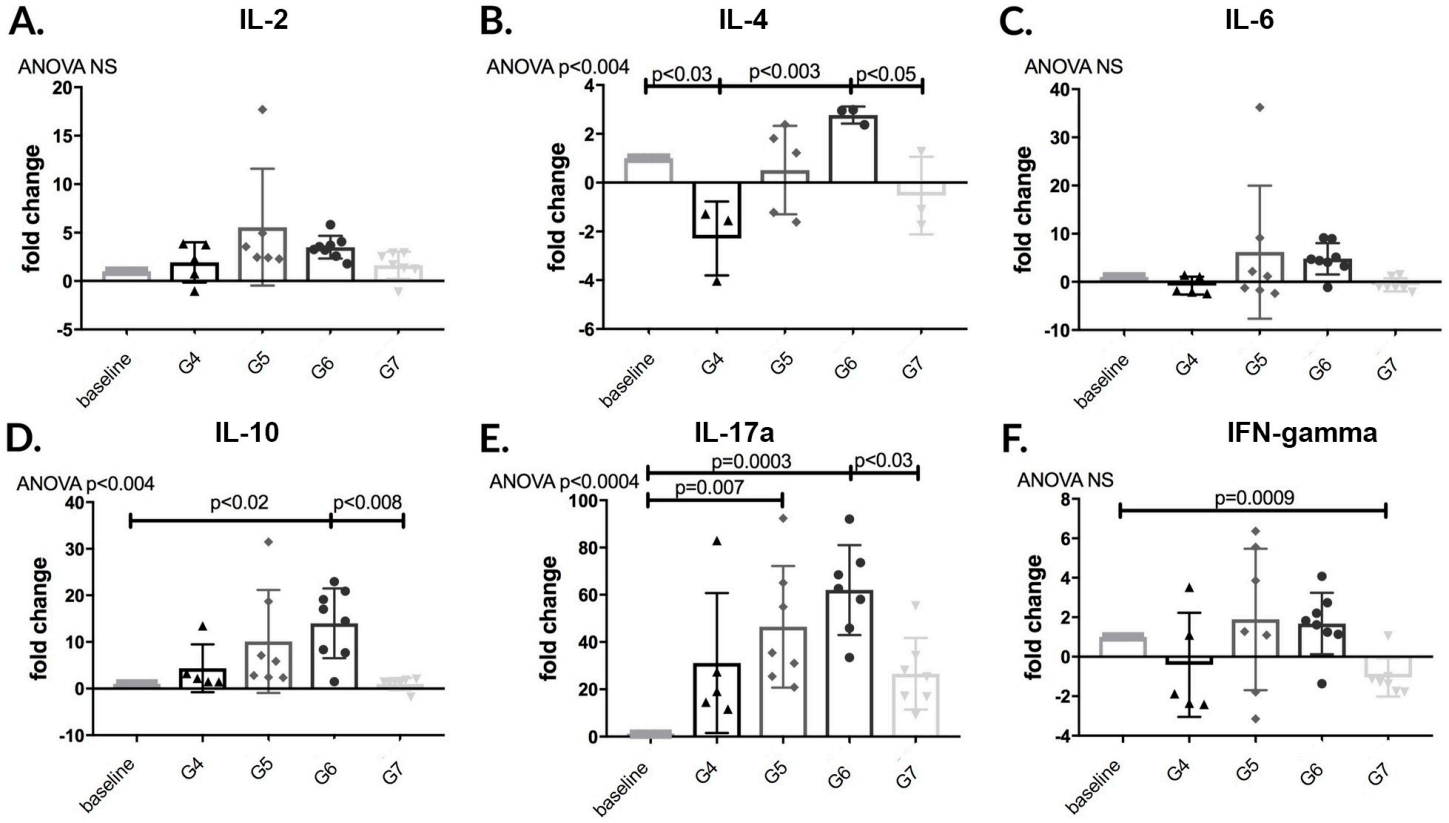
Anti-inflammatory IL-10 significantly increased in post-transplant APRIL/BLyS blockade treated group compared to both pre- post-transplant treated groups. RT-PCR was performed to evaluate changes in cytokine production. (A) IL-2. (B) IL-4. (C) IL-6. (D) IL-10 (E) IL-17a. (F) Interferon-gamma.

Finally, we performed RT-PCR on RNA purified from frozen spleen tissue in order to determine if changes in B T lymphocyte populations subsequent ABMR or ACMR was the result of changes in cytokine production. The anti-inflammatory cytokine IL-10 was found to be significantly increased in G6 animals compared to G7 (p<0.008). IL-17a, a pro-inflammatory mediator linked to several autoimmune diseases, was found to be significantly elevated in G6 animals compared to G7 (p<0.03).[23] No changes were seen in IL-2 or IL-6 production between any groups (Fig 17).

## Discussion

In this study we demonstrated that blockade of APRIL BLyS in a sensitized rodent model results in significant changes in B lymphocyte antibody secreting cell populations. The application of TACI-Ig (APRIL/BLyS blockade) administered for 21 days after sensitization resulted in a consistent decrease in mature B lymphocyte subsets in spleen, bone marrow lymph node IgM spleen bone marrow IgG secreting cells when compared to sensitized untreated animals. Despite this significant reduction in antibody secreting cells, APRIL/BLyS blockade was found to only decrease CD3^+^IgG2a in a flow crossmatch compared to the sensitized untreated group.

Additionally, we explore the effects of APRIL BLyS blockade in a preclinical rodent ABMR kidney transplant model. Transplanted animals were sensitized for 21 days subsequently received APRIL/BLyS blockade prior to transplant for 21 days (G5), after transplant (G6), or both before after transplant (G7). Statistical calculations to determine power were determined prior to implementation of this experiment. G6 animals not only demonstrated no diffuse C4d deposition with minimal microcirculatory inflammation, but no animal developed ABMR. In comparison, 83% of G5 animals 57% of G7 developed ABMR. These findings are consistent with the depletion of germinal center B lymphocytes antibody secreting cells seen in G6 animals when compared to G5. This decrease in antibody secreting cells was not found to have an effect on DSA production. Additionally, reduced ABMR found in animals who received post-transplant APRIL/BLyS blockade (G6) did not correspond with a decrease in DSA. DSA in G6 did trend towards being equal to or reduced compared to other groups; however, this was not statistically significant. Future investigations will continue to examine this result.

APRIL/BLyS blockade demonstrated its ability to deplete plasma cells, IgM IgG secreting cells mature B lymphocyte populations in a rodent model. However, these results were not replicated throughout all groups that received therapy in conjunction with kidney transplant actually resulted in a worsening of ABMR. This finding indicates that timing of APRIL/BLyS administration in relation to transplant plays a significant role in the remodeling of B T lymphocyte compositions.

The impressive level of rejection noted in the groups receiving APRIL/BLyS blockade pre-transplant (G5) both pre- post-transplant (G7) may be related to regulatory B lymphocyte depletion. Regulatory B lymphocytes, initially described in mice later in humans, are found to mediate their function through IL-10.[24] Additionally, plasma cells are found to be a major source of IL-10 IL-35, both of which are known to suppress the differentiation function of pro-inflammatory cells.[25, 26] Mice with IL-35 deficient B lymphocytes were found to lose their ability to recover from EAE (a murine model of MS) due to increased activation of effector T lymphocytes enhanced antigen presentation by B lymphocytes.[27] These cells may potentially play a role in transplant tolerance due to the mounting evidence of a B lymphocyte signature in tolerant transplant recipients.[28, 29] As certain B lymphocyte subsets have demonstrated regulatory or tolerant functions, it is important to weigh the potential advantages disadvantages when depleting B lymphocytes indiscriminately.

Of important note similar to our findings, the timing of B lymphocyte depletion was found to have drastically different outcomes in a murine EAE model. Early B lymphocyte depletion prior to the induction of disease resulted in a significantly more severe disease course compared to animals that received depletion 1–3 weeks following disease induction. Symptomatic improvement following B lymphocyte depletion after disease induction likely was the result of lack of T lymphocyte activation from B lymphocytes either through B cell antigen presentation, B cell co-stimulation or cytokine production which significantly increases T cell activation. Furthermore, in this study by Matsushita et al., the adoptive transfer of B10 regulatory cells, the only B lymphocyte population found to secrete IL-10, resulted in a normalization of EAE pathogenesis. As a result, early depletion of B lymphocytes prior to disease induction was thought to be specifically related to the absence of regulatory B cells.[30]

The role of B lymphocytes as inhibitory cells is further supported in transplant autoimmune literature. Specifically, B lymphocytes are thought to exert their inhibitory function through their interaction with T lymphocytes. In humans, B lymphocyte depletion with rituximab has been shown to worsen ulcerative colitis to cause the development of psoriasis, which indicates B lymphocytes may play an inhibitory role in these primarily T lymphocyte mediated disease processes.[31, 32] Clatworthy et al. examined the effect of B lymphocyte depletion through rituximab in ACMR; however, the study was forced to stop recruitment due to increased rates of ACMR in the treatment group. This was thought to potentially be due to pro-inflammatory cytokine release associated with B lymphocyte depletion, which subsequently resulted in the priming of antigen presenting cells.[33] However, as seen in Mashsushita et al., more prevalent ACMR could also be due to depletion of regulatory B lymphocytes before disease induction.

The immunoregulatory function of B lymphocytes has also been demonstrated through the discovery of a B lymphocyte signature associated with tolerance. Newell et al. found 3 genes expressed during the transition of B lymphocytes into mature B cells or activated B cells. Specifically, increased total B lymphocytes including naïve B cell non-switched memory cells were found in tolerant individuals. The discovery of a tolerant signature involving B lymphocytes further supports the immunoregulatory function of B lymphocytes.[29, 34]

Kwun et al. also used APRIL/BLyS blockade via TACI-Ig in a non-human primate ABMR model as a method to prevent alloantibody production increase graft survival.[35] Here, APRIL/BLyS blockade at the time of transplant was found to decrease peripheral B lymphocyte populations, but more importantly, APRIL/BLyS blockade resulted in decreased DSA post-transplant reduced the histological findings associated with ABMR. Although post-transplant APRIL/BLyS blockade in our model did not decrease DSA despite the decrease in antibody secreting cells, it did result in a lower incidence of ABMR compared to other groups as seen in the Kwun et al. model. Similar to our findings, isotype switching B lymphocytes T lymphocyte populations were not affected by APRIL/BLyS blockade.

TACI-Ig has previously been investigated in the autoimmune literature for treatment of systemic lupus erythematosus (SLE) rheumatoid arthritis.[12, 36] In an SLE trial, APRIL BLyS blockade via atacicept (TACI-Ig) demonstrated a significant reduction in serum IgG levels which supports the idea that APRIL/BLyS blockade via TACI-Ig is able to successfully target plasma cells.[37] Gross et al. utilized TACI-Ig in a murine model of collagen-induced arthritis with treatment given three times weekly for three weeks either before or during the onset of disease. Both groups were found to have a decrease in disease severity anti-collagen antibody production.[12] TACI-Ig treated mice had reduced levels of mature B lymphocytes immunoglobulins. Although disease onset was delayed in the group receiving prophylactic treatment, disease severity increased once treatment stopped at day 21. This supports our data that animals who only received treatment before transplant (but not after transplant) had significantly worse ABMR compared to those receiving treatment after transplant. Dall’Era et al. conducted a multicenter, phase Ib clinical trial, which investigated the effect of TACI-Ig in patients with SLE. They also found a reduction in immunoglobulin levels in mature B lymphocytes. In this study, TACI-Ig was administered after the onset of disease, patients who received two doses had a sustained response. These studies in addition to our results suggest that TACI-Ig is able to effectively deplete B lymphocytes immunoglobulins in autoimmunity transplant; however, the timing duration of treatment is an important factor to consider.

BLyS has previously been implicated in acute chronic ABMR specifically in C4d positive renal transplant biopsies that had acute rejection.[38] Multiple pharmacologic therapies such as belimumab atacicept have been used to target BLyS. Belimumab is a humanized monoclonal antibody that binds neutralizes BLyS has been effective in SLE treatment.[39] Of note, however, naïve B cells are more susceptible to BLyS inhibition in comparison to memory B cells indicating that BLyS blockade may be more beneficial to target dnDSA in patients rather than highly sensitized patients who have large circulating pools of memory B cells.[22, 28] As seen in the present study, APRIL/BLyS blockade did not consistently decrease memory B cells in sensitized non-transplanted or transplanted groups.

### Limitations

The primary limitation of this study is the lack of ABMR in control transplant animals. The rodent model of ABMR in kidney transplantation has previously been established in this model, rodents were sensitized for 3 weeks prior to kidney transplant then harvested 7 days after transplant.[14] These animals developed significant ABMR as evidenced in their Banff classification of kidney histology. The control group used in this study is a modification of that model in order to more closely mirror the experimental groups, which would be receiving APRIL/BLyS blockade for 3 weeks following transplant. It is possible that these control transplant animals did in fact develop ABMR, however, over time the evidence of rejection diminished.

Lastly, the TACI-Ig used in this study was optimized for a mouse sequence rather than a rat sequence. Although these proteins are related in mice rats, there appears to be a difference in efficacy of the drug between these two species due to the fact that we were unable to completely reduce B lymphocytes DSA in rats with TACI-Ig Although the rat mouse ligs are related but dissimilar, we compensated for this fact by performing a dose response titration of the drug as measured by flow crossmatch.

## Conclusions

APRIL/BLyS blockade post-transplant in a preclinical ABMR model has demonstrated a significant decrease in antibody secreting cells, more importantly, in a lower incidence of ABMR compared to groups receiving treatment before both before after transplant. This increase in ABMR seen with pre-transplant treatment is likely related to the depletion of regulatory B lymphocytes that otherwise function to inhibit inflammatory processes including rejection. The ability of APRIL/BLyS blockade to inhibit DSA production, decrease the incidence of ABMR, to decrease the severity of autoimmune disease has been supported in literature as previously discussed. However, the novelty of our study lies in the fact that we have shown that timing of APRIL/BLyS blockade plays a significant role in the development of ABMR when given prior to the development of disease without T lymphocyte inhibition. Future studies will examine the effect of APRIL BLyS inhibition in a genetically modified rodent model.

## Supporting information

S1 Fig No changes seen in non-switched or switched B lymphocytes with APRIL/BLyS blockade. Flow cytometry was used to assess mature B lymphocyte populations for each group. For all rows, left to right: spleen, bone marrow, lymph node, PBMC. (A) Non-switched B lymphocytes were defined as IgD^+^CD45R^+^CD27^+^. (B) Switched B lymphocytes were defined as IgD^-^CD45R^+^IgM^-^CD27^+^.
(TIF)

S2 Fig No significant difference in immature B lymphocyte populations between treatment groups. Flow cytometry was used to assess immature B lymphocyte populations for each group. For all rows, left to right: spleen, bone marrow, lymph node, PBMC. (A) Transitional zone (TZ) B cells were defined as IgD^+^CD45R^+^CD38^+^CD24^+^. (B) Naïve B cells were defined as IgD^+^CD45R^+^CD27^-^.
(TIF)
