# Supplementary figures and images for: Desensitization and treatment with APRIL/BLyS blockade in rodent kidney transplant model
Source: PLoS One. 2019 Feb 8;14(2):e0211865. doi: 10.1371/journal.pone.0211865 (PMC6368307; doi:10.1371/journal.pone.0211865)

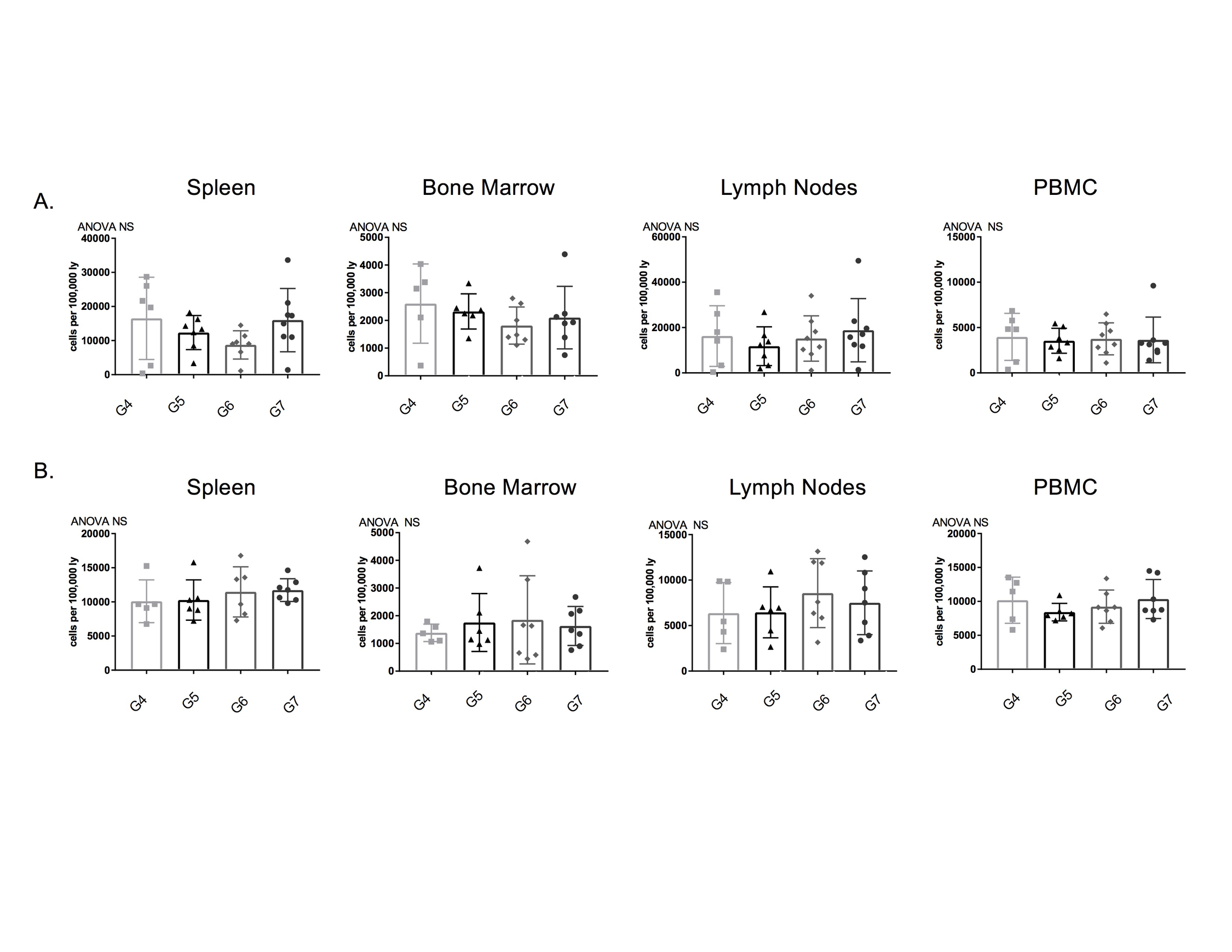

Supplement: S1 Fig — Flow cytometry was used to assess mature B lymphocyte populations for each group. For all rows, left to right: spleen, bone marrow, lymph node, PBMC. (A) Non-switched B lymphocytes were defined as IgD+CD45R+CD27+. (B) Switched B lymphocytes were defined as IgD-CD45R+IgM-CD27+. (TIF) [file pone.0211865.s001.tif]
